# Pathological Pd-phenanthroline complex under standard DFT protocols

**DOI:** 10.1007/s00894-026-06807-3

**Published:** 2026-06-11

**Authors:** Artur Brotons-Rufes, Simona Russo, Doaa R. Ramadan, Manar Ahmed Fouad, Francesco Ferretti, Fabio Ragaini, Chiara Costabile

**Affiliations:** 1 Dipartimento di Chimica e Biologia “A. Zambelli”, Università di Salerno, V. Giovanni Paolo II, Fisciano, SA Italy; 2 Chemistry Department, Faculty of Science, Alexandria University, P.O. Box 426, Alexandria, 21321 Egypt; 3 Dipartimento di Chimica, Università di Milano, V. C. Golgi 19, Milan, Italy

**Keywords:** DFT mechanism, Computational artefacts, Modelling protocols, Palladium, Phenanthroline

## Abstract

**Context:**

The reliability of density functional theory (DFT) calculations for planar π-extended palladacycles can be strongly affected by the modelling of the surrounding chemical environment. In the present study, standard gas-phase calculations lead to an artificial folding of the complex, initially attributed to an overestimation of non-covalent interactions with aromatic additive species. Although improved dispersion treatments partially mitigate the distortion, the introduction of strongly donating ligands exacerbates the curvature even when modern D4 dispersion corrections are employed. The results indicate that the structural deformation does not originate solely from dispersion effects but rather from an artefact associated with the unrealistic empty space opposite the coordinating ligands in the gas-phase model. Importantly, the explicit inclusion of a single solvent molecule substantially reduces the curvature of the palladacycle while maintaining the same dispersion treatment. These findings highlight the importance of minimal yet chemically meaningful environmental models for obtaining physically consistent structures in computational studies of palladium complexes.

**Methods:**

Geometry optimisations were carried out with the Gaussian 16 package, using the BP86 functional with the Def2SVP basis set for the light atoms and SDD for the metal centre with the Stuttgart/Dresden ECPs. Furthermore, dispersion corrections were introduced with Grimme D3 and D4; ORCA 6 software was used for the latter. The energies were refined via single-point calculations on optimized structures using the Def2TZVPP with diffuse basis set for the electronegative atoms, testing dispersion effects on M06 and MN15 functionals, with the double hybrid B2PLYPD3 as reference. Solvent effects were introduced via implicit solvent model (PCM, toluene).

**Supplementary Information:**

The online version contains supplementary material available at 10.1007/s00894-026-06807-3.

## Introduction

1,10-phenanthroline is a well-known molecule, popular for its high coordinative affinity with a wide range of metals, introducing chelation and polyaromaticity into the system, with the additional advantage of being easily modifiable at the 2 to 9 positions [1–3]. For these reasons it has become ubiquitous in almost every modern field of chemistry and material science disciplines [4–6].

**Fig. 1:**
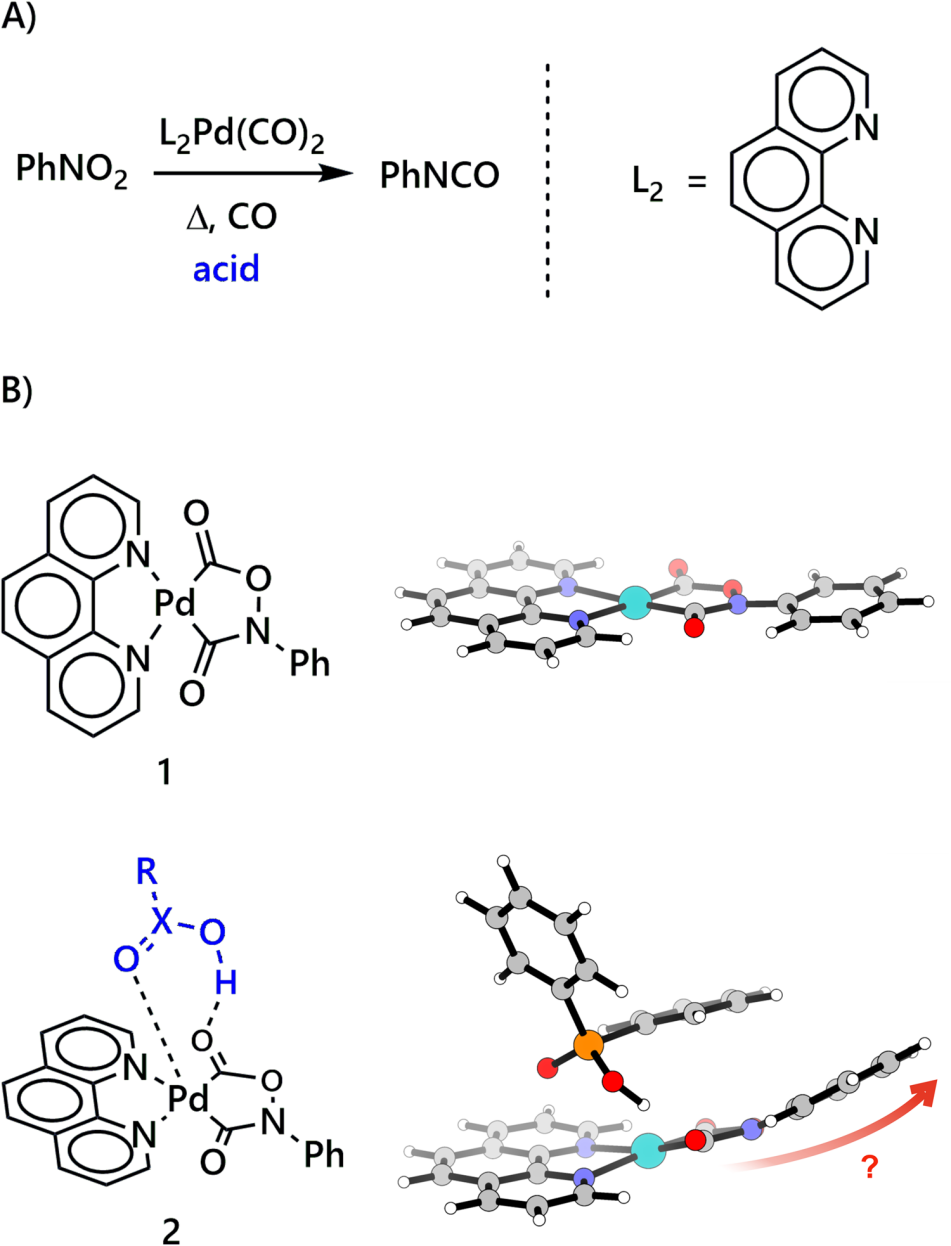
(**a**) Direct isocyanate phosgene-free synthesis with palladium phenanthroline system. (**b**) Key structures of the study, with **1** being the postulated resting state of the process and **2** being the coordination of the acid to it. On the right, respective optimized geometries with the model **uBP86/Def2SVP(H,C,N,O,P) + sdd(Pd)** with Grimme’s D3

In the context of organometallic catalysis, phenanthroline is widely used in palladium redox processes, being well-suited for the square planar geometry of ${d}^{8}$ complexes [7]. More specifically, as part of our long-standing interest in the carbonylation reactions of nitroarenes, [8–11] our group has recently focused its efforts on studying cleaner methods for aryl isocyanate synthesis (see Fig. 1a). Organic isocyanates are critical intermediates for the synthesis of several industrial products, as pesticides and polyurethanes. The most employed aromatic isocyanates are produced nowadays by the reduction of nitroarenes to anilines. The latter are then reacted with phosgene, well known for its toxicity and the dangers it poses to the environment and to those exposed to it. As a green alternative synthesis, many efforts have been devoted in the past decades to the direct carbonylation of nitroarenes, although this approach still faces several problems [12–14].

Very recently, some of us showed that the use of a palladium/phenanthroline catalytic system activated by phosphorus acids, especially diphenyl phosphinic acid, is able to increase the turnover number for this reaction by about one order of magnitude [15]. In the attempt to define the involved mechanism, DFT investigations were performed. Indeed, computational chemistry is an invaluable tool for the rational design of catalytic systems.

Although DFT investigations on this very complicated mechanism are still ongoing, some general outcomes, relative to the modelling of square planar metal complexes featuring π extended delocalization on ligands, were observed.

Consequently, we eventually focused our efforts on evaluating the decomposition of a key palladacycle intermediate **1** (Fig. 1b). Without going into mechanistic details, the coordination of an acid molecule is believed to be necessary to promote the ring opening of the metallacycle **2** (see Fig. 1b).

Herein, we present a short report on a very specific case where the standard DFT protocols for organometallic species yielded an artificial outcome on the optimized geometry. After some tests, we offer a potential explanation and we propose how to mitigate the undesired outcome, while maintaining a low computational cost. The reported final cheap protocols can in principle be extended to several square-planar metal complexes with ligands presenting π delocalized electrons, interacting with other aromatic external groups, as for example commonly reported for many supramolecular systems [16, 17].

## Methods

All optimizations were done with the Gaussian 16 package, except for the ones containing the Grimme’s D4 dispersion correction, for which ORCA 6 was used. Single-point corrections were carried to refine energy and include solvent effects. The exact model for each geometry is specified at the description of each image. Characterization of the minima was done by means of frequency analysis.

## Results and discussion

The study started with a benchmark on **1**, performed with Gaussian16 [18] to select the functional for the geometry optimization. However, the reported crystal structure of **1**, [19] shows a clear packing distortion relative to the phenyl group, that is the folding of the ring out of plane, changing the hybridization of the palladacycle’s nitrogen, leading to the formation of a dimeric assembly of palladacycles through non-covalent interactions (NCI). Note that in all gas optimizations the full planar geometry depicted in Fig. 1a, which fits with a superior delocalization framework, was obtained. Thus, in the absence of a valid XRD, a pairwise comparison of the geometries optimized with different functionals was carried out: BP86 [20, 21], BMK [22], B3LYP [20, 23], CAM-B3LYP [24], PBE0 [25, 26] and, TPSSH [27, 28]. The quantitative comparison of the geometries was done by means of the Chemcraft software’s *Structure Comparer* Tool, which implements a readily available RMSD algorithm (details in SI, *RMSD Computation Details* section). The obtained root mean square deviation (RMSD) values in Table 1 show that the structural parameters are broadly consistent across the tested functionals, with deviations ranging from approximately 0.006 to 0.038. The closest agreement was observed between CAM-B3LYP and PBE0, with BP86 and TPSSH producing relatively similar structures. B3LYP showed the largest deviations from the other functionals, particularly relative to PBE0 and CAM-B3LYP.

**Table 1:** RMSD on the paired functionals used for the optimization geometries of intermediate **1**, in Å

	BP86	B3LYP	CAM-B3LYP	BMK	PBE0	TPSSH
BP86	0.000					
B3LYP	0.026	0.000				
CAM-B3LYP	0.029	0.034	0.000			
BMK	0.029	0.032	0.023	0.000		
PBE0	0.030	0.038	0.006	0.023	0.000	
TPSSH	0.015	0.020	0.018	0.020	0.021	0.000

Finally, BP86 was chosen as the working functional based on practical and structural considerations. BP86 is a pure GGA functional and is therefore computationally less demanding than the hybrid functionals considered here. It also avoids fixed exact-exchange contributions, which is useful for future multiplicity-dependent studies where the role of exact exchange may be introduced and examined separately. The pairwise structural comparison indicates that the BP86-optimised geometry is not an outlier among the tested methods, supporting its use as a consistent reference geometry for further calculations.

**Fig. 2:**
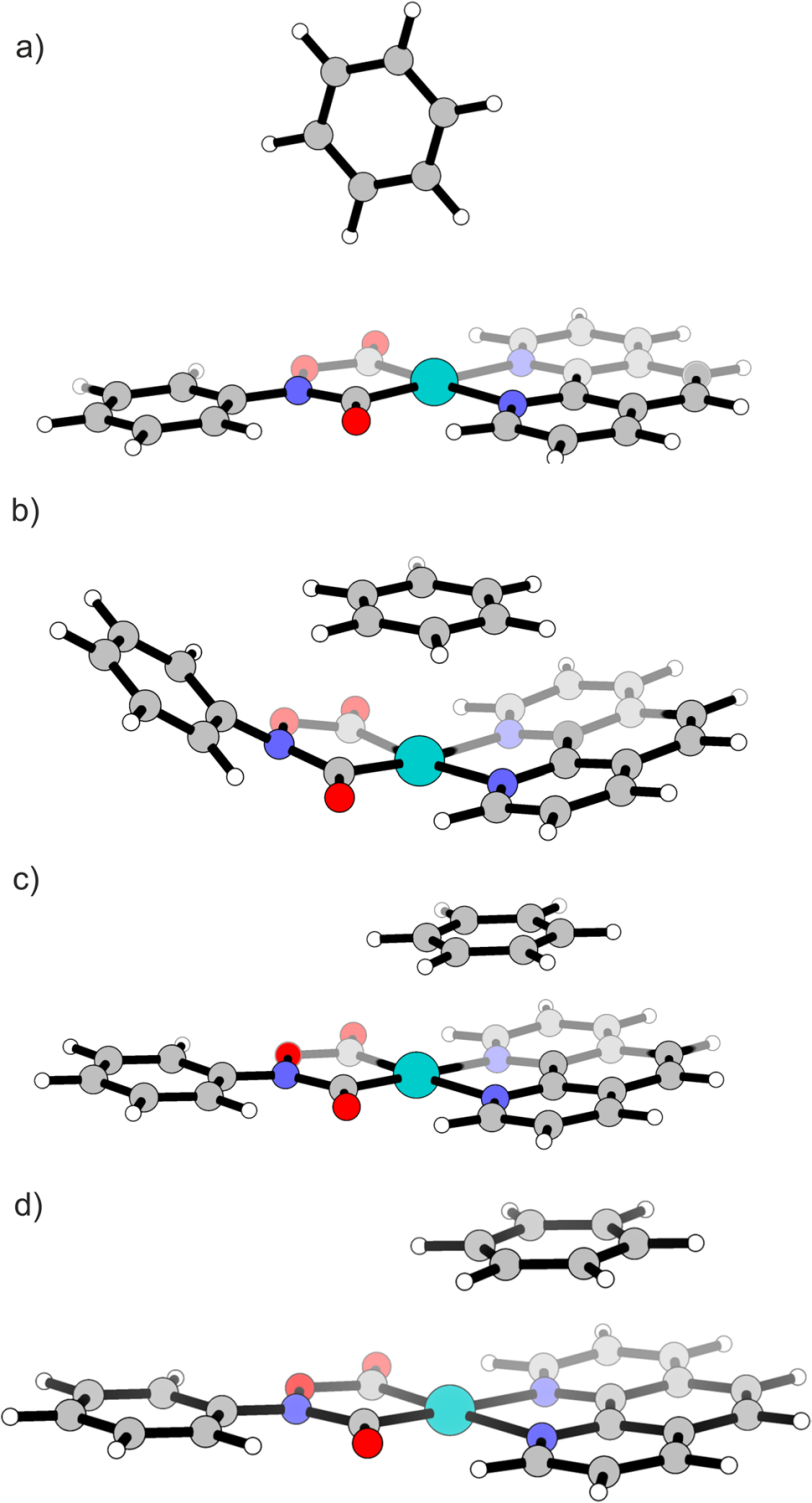
Benzene test before (**a**) and after (**b**) the optimization with D3, with Becke-Johnson damping function (**c**), and with D4 dispersion (**d**). Via model **uBP86/Def2SVP(H,C,N,O,P) + sdd(Pd)**, all ran with ORCA 6.0

Several modifications have been evaluated computationally, for both the assisting acid and the catalyst. Initially, benzoic and dimethyl-phosphinic acids were evaluated, giving rise to several configurations. However, when testing the diphenyl phosphinic acid, an unrealistic folding of the metallacycle appeared (see **2** twisted structure in Fig. 1b). This raised concerns about a potential overestimation of the π−π stacking interactions, an issue widely reported for the classic Grimme D3 dispersion [29]. Some tests were carried out using a benzene molecule, whose small polarization strength should not trigger any curvature on the metallacycle. In the absence of Grimme D3 dispersion, the metallacycle keeps a planar geometry (Fig. 2a). On the contrary, the benzene triggered the same unrealistic curvature when D3 dispersions were introduced, confirming the overestimation of the π−π stacking in the current model (see Fig. 2b). To further confirm it, the test structure was reoptimized, starting from the same geometry, but with modifications in the dispersion. First, we used the Becke-Johnson damping function (D3BJ) [30], which was specifically designed to address these problematic scenarios (see Fig. 2c).

Separately, a complete change in the dispersion treatment was tested via the standard Grimme’s D4 dispersion correction (D4) (see Fig. 2d) [31, 32], available in ORCA [33, 34]. Notably, the latter also works as a reference, being the current state of the art for dispersion description on DFT calculations. Optimisations with no dispersion, D3 and D3BJ were repeated with ORCA, to ensure a consistent comparison, observing no significant changes. Figure 2 shows all structures optimised with ORCA. Both methods yield a coplanar orientation of both molecules with a much milder curvature of the organometallic bicyclic system. This would confirm the initial concern that the non-covalent interactions (NCI) are being overestimated in the original mode, at least when the diphenyl phosphinic acid is introduced. Since the structure obtained with D3BJ dispersion is practically indistinguishable from that obtained with ORCA’s D4 dispersion correction (RMSD = 0.175), and the difference between the ORCA and Gaussian D3BJ optimisations is negligible (RMSD = 0.021), the calculations were kept with the D3BJ to use the more familiar Gaussian 16. 

**Fig. 3:**
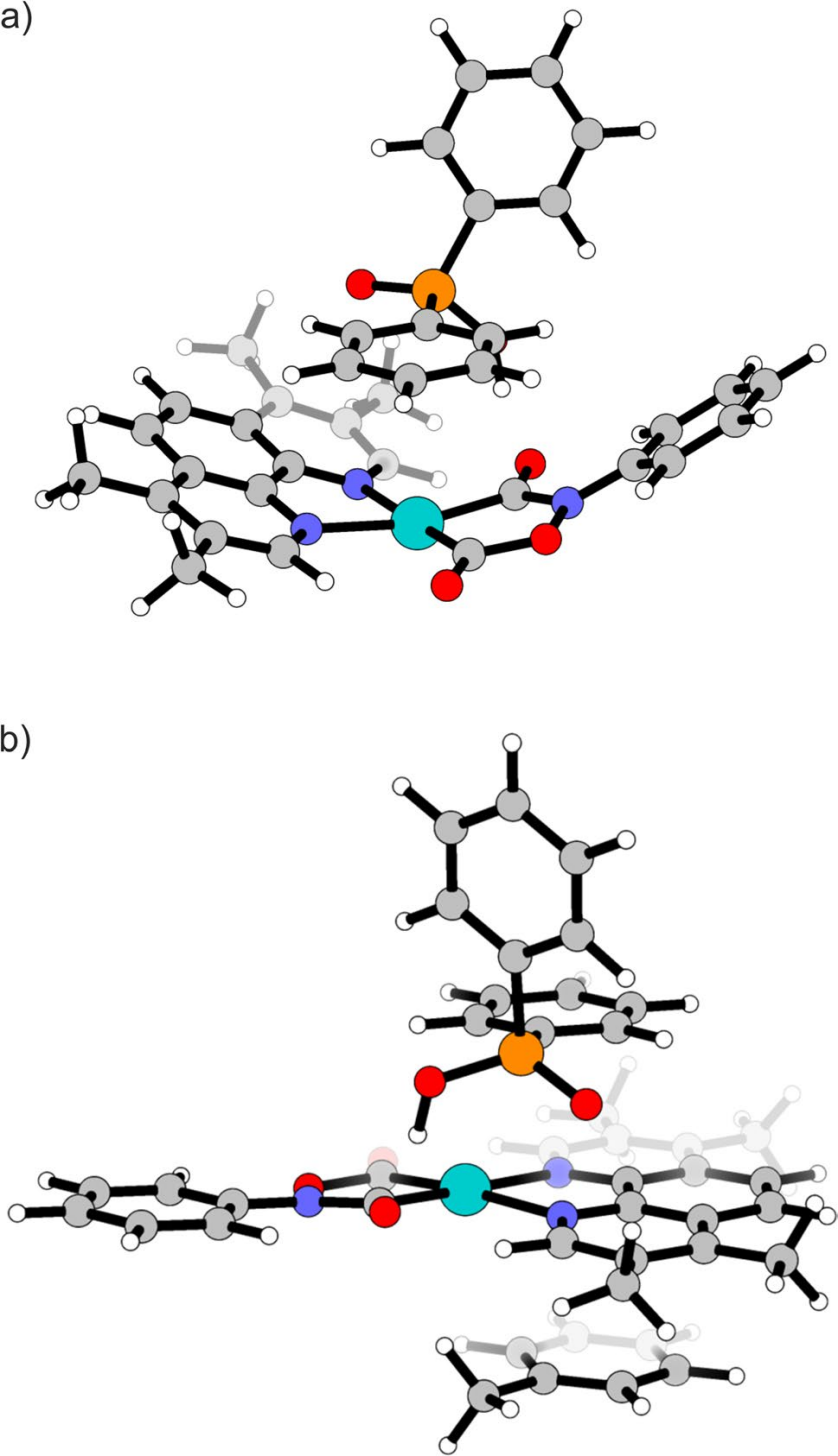
Optimized acid and 4-methyl phenanthroline system, **a**) optimized with the acid on top (done with ORCA’s with Grimme’s D4 dispersion correction (D4), **uBP86/Def2SVP(H,C,N,O,P) + SDD(Pd)** level) and **b**) with an explicit solvent molecules in the opposite side (done with Gaussian 16 and Becke-Johnson damping function at **uBP86/Def2SVP(H,C,N,O,P) + SDD(Pd)** level)

From this point, the computational study continues with the D3BJ dispersion. However, while evaluating electron-donating effects on the 3,4,8,9-tetramethyl-1,10-phenanthroline, the folding problem reappeared. This time, however, even the standard D4 dispersion correction yielded a completely curved structure (see Fig. 3a).

At this point, the possibility was considered that the problem originated from something other than a simple overestimation of the dispersion forces. In this regard, we noted the planarity of the palladacycle **1** in the absence of the additive, which renders it similar to a surface-like system (i.e. quasi-2D electronic structure). Under this assumption, the addition of the acid on a single site of the palladacycle would generate an unrealistic ‘naked’ space on the opposite side. This might generate a strong artificial polarization or gradient that induces the optimization to move towards the observed folding.

**Fig. 4:**
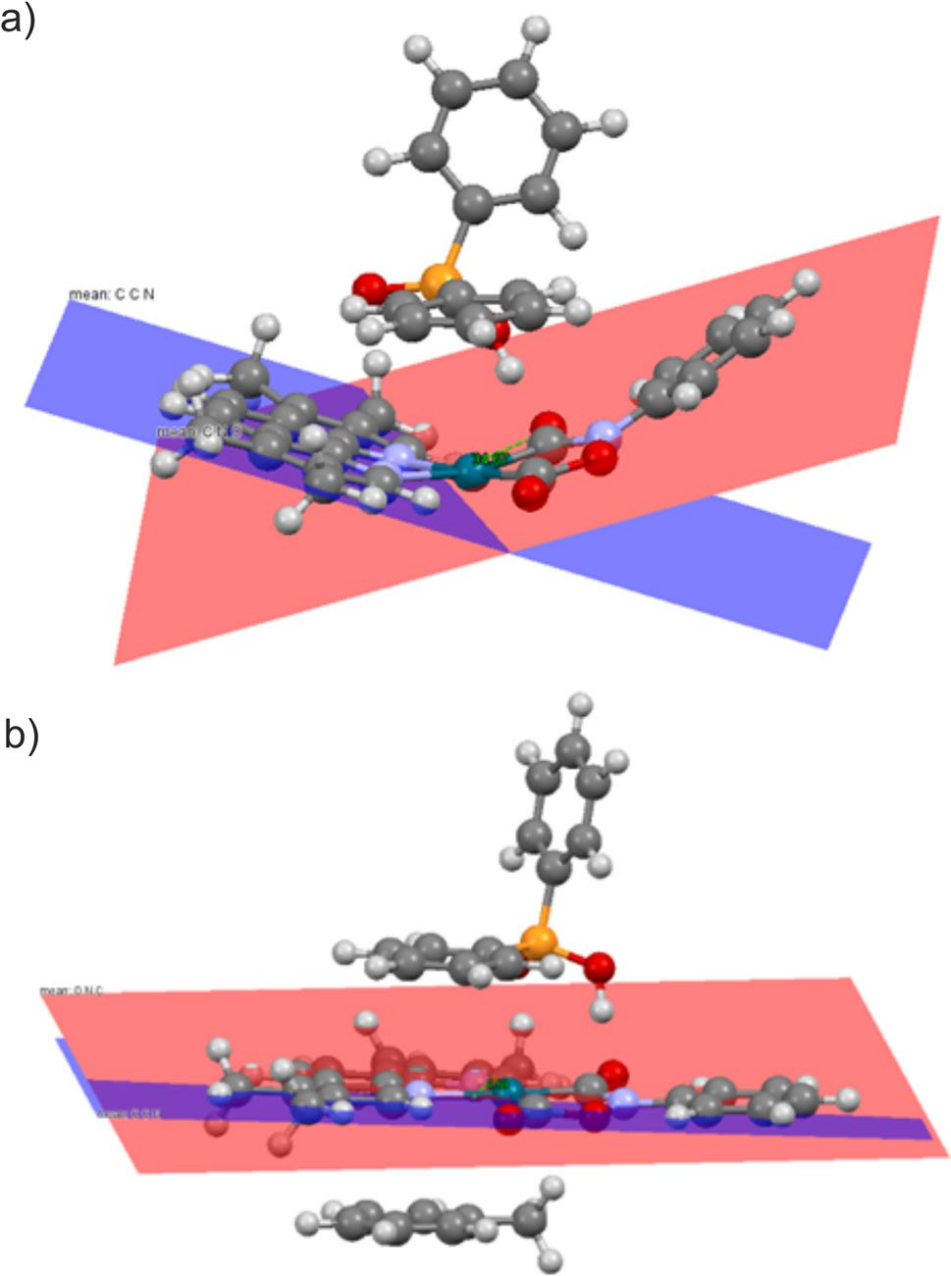
Angle between planes only with the coordinated acid (top, 34.57°), and with the explicit toluene (bottom, 8.56°). Images and planes computed with the Mercury CCDC program [35]

**Fig. 5:**
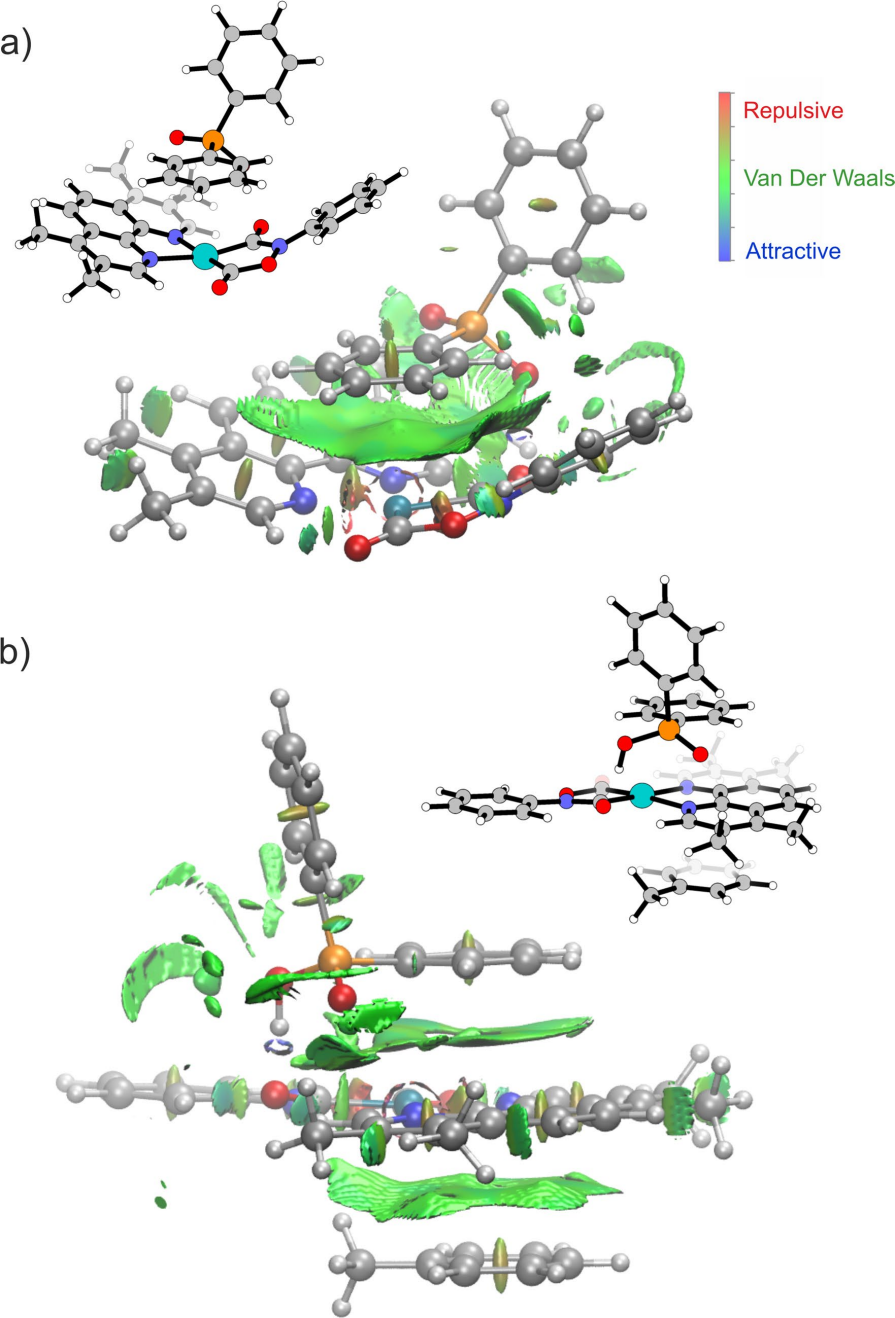
Interaction analysis showing the reduced-density gradient surfaces between the palladacycle and the weakly bonded molecules. Computational details and additional views in the Supplementary Information

To test this hypothesis, two additional tests were performed. First, the optimization was repeated using an implicit solvent model (PCM, toluene), with the intention of mitigating the polarization on the two sides of the complex. However, this did not lead to any noticeable improvement. Second, the optimization was repeated with a toluene on the opposite side of the acidic moiety to approximate explicit solvent effects. The toluene molecule approached the metal complex due to dispersion effects, and a significant reduction of the curvature was observed. Specifically, the angle between the phenanthroline palladacycle and the nitroso-side palladacycle goes from 34.57° to 8.56° (Fig. 4), for the geometries shown in Fig. 3a and b, respectively. Moreover, the influence of additional solvent molecules was examined (cartesian coordinate geometries have been reported in the Supplementary Information). In all cases, the palladacycle remained essentially planar, with no substantial changes in the acid coordination mode. This reinforces the interpretation that the folding observed in the simplified palladacycle model is induced by the artificially empty cavity, rather than representing an inherent structural feature of the full system. In addition, an evaluation of the intramolecular interactions was carried out for both cases. Indeed, a surface of weak interactions can be observed when either the acid or a solvent molecule gets close to the palladacycle moiety (see Fig. 5 and Fig. S1). Including the explicit solvent balances the interactions on both sides, thereby preventing the optimization from causing the unrealistic folding. Along these lines, the interactions between the complex and the acid decrease upon the introduction of toluene, as can be seen by a reduction of the green surface between the first two when going from distorted to planar geometry.

### Dispersion in energy refinement

As is customary in computational chemistry, single-point corrections were performed to refine the energy by using higher-level functionals and basis sets, and to introduce solvent effects. Note that while geometry cannot change due to the nature of the single-point calculations, the significant stacking of the palladacycle and acid moieties makes the final energy potentially susceptible to dispersion effects. Thus, the impact of the dispersion on these steps was also evaluated.

The M06 functional was initially selected due to its significant success in energy and mechanistic descriptors [36]. The broad parametrisation of the M06 functional allows for an implicit description of short-range non-covalent interactions. However, it is also common practice to include additional dispersion to account for a broader variety of related interactions. Since D3BJ dispersion is not parametrisation for M06, the standard D3 was used for the different optimized conformers of the metallacycle and the acid additive. These conformers consist of different binding modes (see Supplementary Information). When comparing the obtained energies, we saw that the addition of dispersion is not only relevant, but the relative stability of the conformers is inverted depending on the degree of dispersion included (see Table 2).

**Table 2:** Relative free energies of **2** conformers with respect to intermediate **1**, using different functionals list on top

Species	$\mathrm{M}0{6}_{\mathrm{n}\mathrm{o}\mathrm{D}}$^a^	$\mathrm{M}0{6}_{\mathrm{D}3}$	$\mathrm{M}\mathrm{N}15$	$\mathrm{B}\mathrm{P}2\mathrm{L}\mathrm{Y}\mathrm{P}\mathrm{D}3+\mathrm{M}\mathrm{P}2$
**1**	0.0	0.0	0.0	0.0
**2**	0.6	− 5.7	− 4.3	− 7.15
${2}_{\mathrm{c}\mathrm{o}\mathrm{n}\mathrm{f}1}$	0.8	− 5.3	− 3.8	− 6.08
${2}_{\mathrm{c}\mathrm{o}\mathrm{n}\mathrm{f}2}$	5.7	0.5	1.7	− 1.19
${2}_{\mathrm{c}\mathrm{o}\mathrm{n}\mathrm{f}3}$	2.5	− 1.1	0.1	− 2.01
${2}_{\mathrm{c}\mathrm{o}\mathrm{n}\mathrm{f}4}$	5.1	− 0.4	0.8	− 3.14

^a^noD denotes calculations performed without additional empirical dispersion corrections

Values in kcal/mol

In order to assess whether the additional D3 in the single-point calculations was again introducing an NCI overestimation, a brief benchmark was designed. Although the use of post-HF gold-standard, CCSD(T), would have been ideal, the size of the system made it prohibitive. Thus, a double hybrid functional was employed instead by means of the B2PLYPD3 (+ MP2) functional, where MP2 refers to the Møller–Plesset perturbation theory contribution of the double hybrid [30, 43] Note that the latter incorporates the damping function form of D3BJ, and has been reported to better account for NCI because of a more accurate description of electron correlation, mostly thanks to its perturbative inclusion [44, 45]. The single hybrid counterpart of this functional (omitting the MP2 perturbative contribution) was also included in the benchmark for comparison, denoted simply as B2PLYPD3. This version is equivalent to a single hybrid functional, and it is assumed to give less accurate results. Additionally, other functionals were evaluated (see Table 2), like the Minnesota MN15 functional, reported to remarkably succeed with systems of significant dispersion [46]. The relative energy of each conformer with respect to the original intermediate **2** was computed and compared to the relative values obtained with the double hybrid model (via magnitude of the relative errors). The results from the free-energies can be seen in the heat-map below (see Table 3, S3 and S4).

**Table 3:**
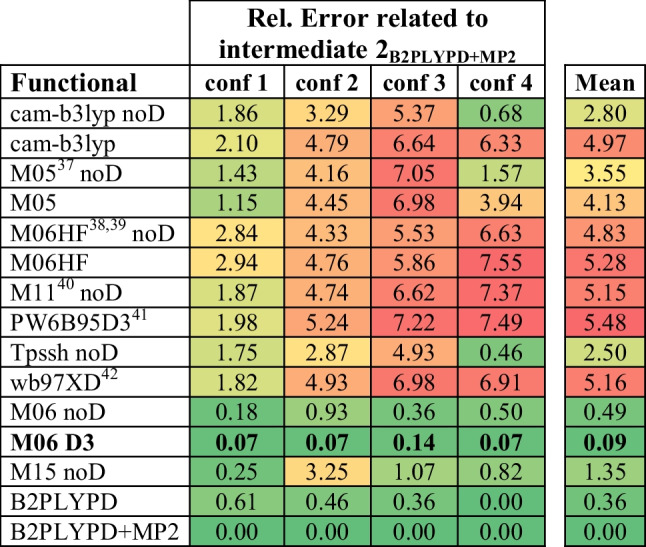
Heat map of the relative errors in conformer free energies (kcal/mol) computed with different DFT functionals, using B2PLYPD3 + MP2 as the reference method

Errors are reported for four conformers relative to the corresponding intermediate structure. The mean absolute error across conformers is shown in the rightmost column. Lower values indicate better reproduction of the reference conformational energy ordering and spacing. Calculations performed without additional empirical dispersion corrections are denoted as noD

M06-D3 showed the lowest mean deviation from the reference, indicating that it provides the best compromise for describing the relative stability of these conformers. B2PLYPD3 (+ MP2) and M06 without dispersion also performed well, whereas several other functionals showed larger deviations, particularly for higher-energy conformers. Surprisingly, the former was the only case where the dispersion rendered smaller error values. For all the other functionals, no dispersion gave better results. The newly obtained relative stabilities were compared with the previous M06 results in Table 2. Although in different degrees, the double hybrid model shows a stabilisation gain of the catalyst-acid conformer with respect to the cycle, **1**. This tendency was reproduced by the M06 functional only with the additional D3 dispersion, although in milder degree. This not only suggests that such dispersion model does not overestimate the NCI between the two fragments of the adduct, but that even with the additional dispersion some are underestimated. Similarly, the MN15 also accounts for the stabilisation of the system, although a bit higher in energy.

Overall, these results highlight the significant complexity of the non-covalent interactions between the palladium complex and an aromatic ring. Such interactions appear to be properly captured only through the perturbative improvement of electron correlation introduced at the double hybrid rung of Jacob’s ladder.

## Conclusion

In summary, DFT calculations, performed on π-extended square planar palladacycles interacting with other π molecular systems, were found to be rather insidious.

Tests performed on a simplified benzene model strongly suggest that the folding observed during geometry optimisations has no genuine chemical origin. Rather, the evidence points to an intrinsic artifact arising from the presence of artificial empty space in the computational model, combined with the extended, quasi-surface nature of intermediate 1. The use of more sophisticated dispersion treatments that mitigate the overestimation of π–π stacking interactions—such as the Becke–Johnson damping function or the D4 dispersion correction—significantly reduces this artifact. However, increasing the electron density exacerbates the distortion.

In cases where this artificial folding affects only a single intermediate along the reaction pathway, we deem it reasonable to explicitly include a solvent molecule at that stage. Indeed, the presence of a solvent molecule restores the environmental interactions missing in gas-phase calculations. This modification fully corrects the distortion, after which the standard computational protocol can be applied to the remainder of the study.

Finally, the inclusion of the dispersion also impacted on the final single-point corrections. Since higher-level calculations were found to be prohibitively expensive and the relative stabilities are largely recovered within the M06 + D3 framework, the following final computational model is proposed:

uM06 ~ GD3/Def2TZVPP(H, C, P) + Def2TZVPPD(N,O) + SDD(Pd) + ECP(SDD)//uBP86 ~ GD3BJ/Def2SVP(H, C, O, N) + SDD(Pd) + ECP(SDD).

## Supplementary Information

Below is the link to the electronic supplementary material.

Supplementary file1 (DOCX 690 KB)

## Data Availability

No datasets were generated or analyzed during the current study.
