# Supplementary material for: Pathological Pd-phenanthroline complex under standard DFT protocols
Source: J Mol Model. 2026 Jun 11;32(7):228. doi: 10.1007/s00894-026-06807-3 (PMC13260194; doi:10.1007/s00894-026-06807-3)
Supplement: Supplementary file 1 — Supplementary file1 (DOCX 690 KB) [file 894_2026_6807_MOESM1_ESM.docx]

Supplementary Information

of

Pathological Pd-phenanthroline complex under standard DFT protocols

Artur Brotons-Rufes,^1^ Simona Russo,^1^ Doaa R. Ramadan,^2,3^ Manar Ahmed Fouad,^2,3^ Francesco Ferretti,^3^ Fabio Ragaini,^3^ Chiara Costabile^1^

Correspondence to: Chiara Costabile (Email: [ccostabile@unisa.it](mailto:ccostabile@unisa.it))

1 Dipartimento di Chimica e Biologia “A. Zambelli”, Università di Salerno, V. Giovanni Paolo II, Fisciano (SA), Italy

2 Chemistry Department, Faculty of Science, Alexandria University, P.O. Box 426, Alexandria 21321, Egypt.

3 Dipartimento di Chimica, Università di Milano, V. C. Golgi 19, Milano, Italy

Summary

[Geometries 3](#_Toc230705950)

[b3lyp_1 (palladacycle alone) with D3_Gaussian 16 SCF Done: -1287.07237906 A.U. 3](#_Toc230705951)

[bmk_1 (palladacycle alone) with D3 Gaussian 16_SCF Done: -1285.71044576 A.U. 4](#_Toc230705952)

[cam-b3lyp_1 (palladacycle alone) with D3 Gaussian 16_SCF Done: -1286.39975230 A.U. 6](#_Toc230705953)

[pbe1pbe_1 (palladacycle alone) with D3 Gaussian16_SCF Done: -1285.70073713 A.U. 7](#_Toc230705954)

[tpssh_1 (palladacycle alone) with D3 Gaussian 16_SCF Done: -1287.03556527 A.U. 9](#_Toc230705955)

[bp86_1 (palladacycle alone) with D3 Gaussian 16 10](#_Toc230705956)

[bp86_2 optimized without any dispersion_Gaussian 16 12](#_Toc230705957)

[bp86 2 optimized with GD3 (Figure 1) (Gaussian 16) 14](#_Toc230705958)

[bp86_(Benzene + palladacycle 1) adduct NO dispersion (Figure 2 a) ORCA 6.0 17](#_Toc230705959)

[bp86_(Benzene + palladacycle 1) adduct D3 (Figure 2 b) ORCA 6.0 18](#_Toc230705960)

[bp86_(Benzene + palladacycle 1) adduct NO dispersion (Figure 2 c) ORCA 6.0 20](#_Toc230705961)

[bp86_(Benzene + palladacycle 1) adduct D4 (Figure 2 d) ORCA 6.0 22](#_Toc230705962)

[bp86_(Benzene + palladacycle 1) adduct D3BJ (Figure 2 d) Gaussian 16 (used for RMSD) 24](#_Toc230705963)

[bp86_4-methyl phenanthroline system with D4 (Figure 3 a) ORCA 6.0 26](#_Toc230705964)

[bp86_ Toluene + 4-methyl phenanthroline system with D3BJ (Figure 3 b) Gaussian 16 29](#_Toc230705965)

[Intermediate $\mathbf{2}\boldsymbol{conf}\mathbf{1}$- used for benchmarks in Table 2 and 3 32](#_Toc230705966)

[Intermediate $\mathbf{2}\boldsymbol{conf}\mathbf{2}$ used for benchmarks in Table 2 and 3 35](#_Toc230705967)

[Intermediate $\mathbf{2}\boldsymbol{conf}\boldsymbol{3}$ used for benchmarks in Table 2 and 3 37](#_Toc230705968)

[Intermediate $\mathbf{2}\boldsymbol{conf}\mathbf{4}$ used for benchmarks in Table 2 and 3 40](#_Toc230705969)

[palladacycle 1 + Toluene on O – side 42](#_Toc230705970)

[palladacycle 1 +Toluene on N – side 46](#_Toc230705971)

[palladacycle 1 + Toluene on N – side 49](#_Toc230705972)

[palladacycle 1 + Toluene on O – side 53](#_Toc230705973)

[palladacycle 1 +Three toluene molecules 56](#_Toc230705974)

[NCI analysis 62](#_Toc230705975)

[Energies 63](#_Toc230705976)

[RMSD analysis 66](#_Toc230705977)

# Geometries

Table S1. Geometries of the discussed structures in the article.

| 40 b3lyp_1 (palladacycle alone) with D3_Gaussian 16 SCF Done: -1287.07237906 A.U. 46 -0.047399000 -0.321413000 -0.000348000  7 -1.472592000 1.358082000 -0.000005000  6 -1.167196000 2.649575000 0.000096000  6 -2.161854000 3.651260000 0.000308000  6 -3.494838000 3.283532000 0.000415000  6 -3.840763000 1.910166000 0.000316000  6 -2.775175000 0.974081000 0.000102000  1 -0.094150000 2.874976000 0.000010000  1 -1.863295000 4.701091000 0.000386000  1 -4.286298000 4.037763000 0.000581000  6 -5.196300000 1.431445000 0.000422000  6 -3.067172000 -0.441640000 0.000000000  7 -2.022159000 -1.307728000 -0.000199000  6 -4.415683000 -0.881535000 0.000111000  6 -2.250241000 -2.615034000 -0.000303000  6 -5.472104000 0.093312000 0.000323000  6 -4.638984000 -2.280034000 0.000003000  1 -6.506292000 -0.259815000 0.000406000  1 -6.006747000 2.164544000 0.000585000  6 -3.558992000 -3.143923000 -0.000207000  1 -1.356832000 -3.249597000 -0.000494000  1 -3.698596000 -4.226397000 -0.000304000  1 -5.663620000 -2.661287000 0.000080000  6 1.688907000 0.644855000 -0.000257000  8 1.808168000 1.863353000 -0.000586000  7 2.785636000 -0.206789000 0.000053000  8 2.511407000 -1.578603000 0.000293000  6 1.154201000 -1.885537000 0.000055000  8 0.875107000 -3.057987000 0.000681000  6 4.170182000 0.075290000 0.000079000  6 5.098846000 -0.984589000 0.000199000  6 6.467986000 -0.713666000 0.000224000  6 6.940643000 0.600972000 0.000132000  6 6.016033000 1.649169000 0.000015000  6 4.642090000 1.404075000 -0.000010000  1 7.171901000 -1.550336000 0.000317000  1 8.013926000 0.805984000 0.000152000  1 6.363993000 2.685810000 -0.000056000  1 3.931329000 2.225288000 -0.000102000  1 4.743891000 -2.012718000 0.000273000  40 bmk_1 (palladacycle alone) with D3 Gaussian 16_SCF Done: -1285.71044576 A.U. 46 -0.066161000 -0.306678000 -0.000075000  7 -1.455387000 1.356989000 0.000005000  6 -1.147336000 2.643384000 0.000017000  6 -2.146436000 3.647479000 0.000074000  6 -3.480082000 3.278356000 0.000119000  6 -3.822458000 1.901033000 0.000103000  6 -2.754276000 0.971763000 0.000044000  1 -0.071596000 2.867786000 -0.000030000  1 -1.847372000 4.698221000 0.000080000  1 -4.273982000 4.032105000 0.000163000  6 -5.181121000 1.415137000 0.000142000  6 -3.040111000 -0.449581000 0.000019000  7 -1.990651000 -1.305063000 -0.000037000  6 -4.383697000 -0.896355000 0.000057000  6 -2.200172000 -2.611068000 -0.000061000  6 -5.450135000 0.075333000 0.000120000  6 -4.597263000 -2.299463000 0.000029000  1 -6.483400000 -0.284425000 0.000149000  1 -5.995620000 2.145658000 0.000188000  6 -3.508429000 -3.153866000 -0.000032000  1 -1.295582000 -3.234582000 -0.000108000  1 -3.636773000 -4.238763000 -0.000057000  1 -5.619983000 -2.689861000 0.000055000  6 1.667202000 0.658480000 -0.000083000  8 1.806011000 1.871645000 0.000041000  7 2.760332000 -0.194817000 -0.000008000  8 2.478378000 -1.560236000 -0.000015000  6 1.139356000 -1.866534000 -0.000082000  8 0.868505000 -3.038806000 -0.000041000  6 4.140587000 0.074398000 0.000001000  6 5.061364000 -0.995839000 -0.000141000  6 6.435287000 -0.737635000 -0.000129000  6 6.922886000 0.573988000 0.000024000  6 6.006000000 1.631758000 0.000169000  6 4.626899000 1.400867000 0.000162000  1 7.130257000 -1.582859000 -0.000241000  1 7.998868000 0.768770000 0.000032000  1 6.362879000 2.666280000 0.000294000  1 3.925230000 2.230967000 0.000272000  1 4.698894000 -2.022489000 -0.000255000  40 cam-b3lyp_1 (palladacycle alone) with D3 Gaussian 16_SCF Done: -1286.39975230 A.U. 46 -0.058145000 -0.316883000 -0.000293000  7 -1.449479000 1.351037000 -0.000122000  6 -1.139613000 2.634276000 -0.000162000  6 -2.129456000 3.635374000 -0.000045000  6 -3.456478000 3.271870000 0.000116000  6 -3.804361000 1.903368000 0.000164000  6 -2.749364000 0.973286000 0.000038000  1 -0.064897000 2.852672000 -0.000297000  1 -1.828584000 4.683590000 -0.000085000  1 -4.245905000 4.027173000 0.000210000  6 -5.160453000 1.426974000 0.000333000  6 -3.042180000 -0.440571000 0.000087000  7 -1.998696000 -1.301646000 -0.000031000  6 -4.379304000 -0.877050000 0.000255000  6 -2.221468000 -2.602767000 0.000007000  6 -5.435592000 0.097582000 0.000376000  6 -4.601111000 -2.271469000 0.000296000  1 -6.468740000 -0.255908000 0.000505000  1 -5.968630000 2.161293000 0.000427000  6 -3.526337000 -3.130917000 0.000171000  1 -1.325134000 -3.233397000 -0.000103000  1 -3.664903000 -4.212602000 0.000196000  1 -5.624924000 -2.652851000 0.000424000  6 1.668347000 0.637297000 0.000059000  8 1.791340000 1.851686000 0.000161000  7 2.758899000 -0.210445000 0.000060000  8 2.485167000 -1.571090000 -0.000132000  6 1.136983000 -1.871809000 -0.000327000  8 0.852256000 -3.038955000 -0.000389000  6 4.139888000 0.072236000 0.000122000  6 5.061452000 -0.984532000 -0.000117000  6 6.425902000 -0.716832000 -0.000058000  6 6.897829000 0.592215000 0.000237000  6 5.978549000 1.637432000 0.000477000  6 4.609217000 1.394536000 0.000425000  1 7.128552000 -1.553346000 -0.000248000  1 7.970474000 0.795784000 0.000282000  1 6.327947000 2.672607000 0.000713000  1 3.896827000 2.213588000 0.000609000  1 4.702710000 -2.010722000 -0.000346000  40 pbe1pbe_1 (palladacycle alone) with D3 Gaussian16_SCF Done: -1285.70073713 A.U. 46 -0.055175000 -0.316354000 -0.000108000  7 -1.448477000 1.345734000 -0.000045000  6 -1.137928000 2.630915000 -0.000058000  6 -2.126155000 3.633533000 -0.000007000  6 -3.457100000 3.272311000 0.000061000  6 -3.806953000 1.903947000 0.000082000  6 -2.747155000 0.970208000 0.000028000  1 -0.061385000 2.847226000 -0.000106000  1 -1.823800000 4.682084000 -0.000020000  1 -4.245334000 4.029800000 0.000103000  6 -5.159079000 1.427517000 0.000156000  6 -3.038414000 -0.440038000 0.000051000  7 -1.994070000 -1.297477000 -0.000002000  6 -4.380929000 -0.879292000 0.000120000  6 -2.215837000 -2.600882000 -0.000001000  6 -5.434581000 0.092695000 0.000173000  6 -4.599496000 -2.274604000 0.000129000  1 -6.468881000 -0.259595000 0.000227000  1 -5.969564000 2.160333000 0.000198000  6 -3.519338000 -3.132343000 0.000065000  1 -1.316702000 -3.229661000 -0.000067000  1 -3.655532000 -4.215067000 0.000062000  1 -5.622672000 -2.659530000 0.000179000  6 1.665547000 0.638209000 0.000081000  8 1.789563000 1.852926000 0.000096000  7 2.754849000 -0.211484000 0.000045000  8 2.483087000 -1.564896000 -0.000126000  6 1.135915000 -1.865763000 -0.000397000  8 0.854512000 -3.034835000 -0.000051000  6 4.130868000 0.070712000 0.000052000  6 5.054943000 -0.987200000 -0.000205000  6 6.420481000 -0.718569000 -0.000203000  6 6.893011000 0.591989000 0.000051000  6 5.971723000 1.637995000 0.000306000  6 4.601414000 1.395175000 0.000313000  1 7.123162000 -1.555862000 -0.000405000  1 7.966136000 0.796049000 0.000051000  1 6.320359000 2.674079000 0.000511000  1 3.887459000 2.214863000 0.000512000  1 4.695787000 -2.014505000 -0.000403000  40 tpssh_1 (palladacycle alone) with D3 Gaussian 16_SCF Done: -1287.03556527 A.U. 46 -0.058884000 -0.318020000 0.000253000  7 -1.455365000 1.346444000 0.000171000  6 -1.143358000 2.639634000 0.000260000  6 -2.133945000 3.644719000 0.000193000  6 -3.471041000 3.285674000 0.000031000  6 -3.824791000 1.913592000 -0.000063000  6 -2.762571000 0.973060000 0.000014000  1 -0.067293000 2.854248000 0.000403000  1 -1.830545000 4.692918000 0.000273000  1 -4.256496000 4.045589000 -0.000022000  6 -5.180292000 1.435033000 -0.000230000  6 -3.054887000 -0.438642000 -0.000079000  7 -2.002562000 -1.298411000 -0.000006000  6 -4.402767000 -0.881762000 -0.000246000  6 -2.227647000 -2.609540000 -0.000095000  6 -5.457664000 0.094604000 -0.000318000  6 -4.620668000 -2.281809000 -0.000335000  1 -6.492470000 -0.255702000 -0.000446000  1 -5.991256000 2.167078000 -0.000288000  6 -3.534807000 -3.141010000 -0.000261000  1 -1.328908000 -3.237435000 -0.000035000  1 -3.670916000 -4.223687000 -0.000330000  1 -5.642619000 -2.669064000 -0.000465000  6 1.672834000 0.643775000 0.000166000  8 1.801155000 1.866257000 0.000014000  7 2.771159000 -0.206240000 0.000116000  8 2.498573000 -1.580526000 0.000146000  6 1.135244000 -1.879096000 0.000172000  8 0.857541000 -3.056276000 0.000307000  6 4.155444000 0.072649000 -0.000080000  6 5.080735000 -0.990461000 -0.000296000  6 6.450777000 -0.720800000 -0.000491000  6 6.925118000 0.594149000 -0.000475000  6 6.001598000 1.644604000 -0.000257000  6 4.626612000 1.402034000 -0.000057000  1 7.153370000 -1.558224000 -0.000658000  1 7.998408000 0.797713000 -0.000627000  1 6.351092000 2.680458000 -0.000238000  1 3.912147000 2.221195000 0.000110000  1 4.719825000 -2.016802000 -0.000308000 |
| --- |
| 40 bp86_1 (palladacycle alone) with D3 Gaussian 16 46 0.061322000 -0.320626000 0.000321000  7 1.444180000 1.348351000 0.000057000  6 1.124118000 2.646896000 0.000018000  6 2.115691000 3.658617000 -0.000154000  6 3.461992000 3.303371000 -0.000288000  6 3.822970000 1.927673000 -0.000250000  6 2.758159000 0.977861000 -0.000072000  1 0.035574000 2.851273000 0.000133000  1 1.806449000 4.714135000 -0.000179000  1 4.251342000 4.071773000 -0.000424000  6 5.182547000 1.450747000 -0.000380000  6 3.054697000 -0.436036000 -0.000026000  7 1.999302000 -1.301249000 0.000143000  6 4.411015000 -0.879407000 -0.000159000  6 2.226469000 -2.619340000 0.000194000  6 5.465140000 0.102683000 -0.000337000  6 4.631306000 -2.284472000 -0.000106000  1 6.509121000 -0.247693000 -0.000438000  1 5.998050000 2.190763000 -0.000517000  6 3.539988000 -3.149570000 0.000072000  1 1.316520000 -3.248515000 0.000355000  1 3.679312000 -4.240566000 0.000124000  1 5.662245000 -2.672527000 -0.000202000  6 -1.670083000 0.645531000 0.000244000  8 -1.802717000 1.875569000 0.000337000  7 -2.769055000 -0.212445000 -0.000050000  8 -2.494431000 -1.595453000 -0.000200000  6 -1.121524000 -1.891202000 0.000052000  8 -0.839785000 -3.075333000 -0.000408000  6 -4.153635000 0.067654000 -0.000087000  6 -5.085640000 -0.998863000 -0.000079000  6 -6.460799000 -0.724263000 -0.000118000  6 -6.933607000 0.598455000 -0.000166000  6 -6.003263000 1.652023000 -0.000177000  6 -4.623191000 1.405019000 -0.000144000  1 -7.171415000 -1.566092000 -0.000111000  1 -8.014723000 0.806270000 -0.000197000  1 -6.353132000 2.696760000 -0.000219000  1 -3.897145000 2.225433000 -0.000144000  1 -4.722620000 -2.033321000 -0.000045000 |
| 66 bp86_2 optimized without any dispersion_Gaussian 16 46 -0.727806000 -1.190668000 -0.435763000  7 0.134522000 -2.670543000 0.896982000  6 -0.406189000 -3.118103000 2.035347000  6 0.290241000 -3.994945000 2.903002000  6 1.573885000 -4.415035000 2.566004000  6 2.165179000 -3.957612000 1.355949000  6 1.393367000 -3.067272000 0.548082000  1 -1.434633000 -2.759595000 2.231474000  1 -0.196227000 -4.334465000 3.829558000  1 2.137004000 -5.099436000 3.220947000  6 3.478808000 -4.347794000 0.909563000  6 1.937388000 -2.569629000 -0.696127000  7 1.168534000 -1.712291000 -1.427041000  6 3.234672000 -2.990976000 -1.120962000  6 1.629698000 -1.259576000 -2.596199000  6 3.990834000 -3.886520000 -0.282476000  6 3.704350000 -2.490807000 -2.366761000  1 4.993862000 -4.197032000 -0.615813000  1 4.067163000 -5.031426000 1.542054000  6 2.897815000 -1.631090000 -3.106047000  1 0.968747000 -0.563430000 -3.138072000  1 3.229906000 -1.225851000 -4.073253000  1 4.700157000 -2.788866000 -2.732369000  6 -2.558597000 -1.057004000 0.319501000  8 -2.981800000 -1.675807000 1.300527000  7 -3.401876000 -0.237654000 -0.442519000  8 -2.783849000 0.484847000 -1.486552000  6 -1.433415000 0.199608000 -1.617706000  8 -0.841598000 0.904161000 -2.442858000  6 -4.783932000 0.043150000 -0.324297000  6 -5.401694000 0.944498000 -1.227108000  6 -6.773593000 1.216674000 -1.115670000  6 -7.553094000 0.608373000 -0.117590000  6 -6.934906000 -0.284276000 0.775375000  6 -5.565276000 -0.574022000 0.685906000  1 -7.235438000 1.919950000 -1.827357000  1 -8.629287000 0.827047000 -0.036299000  1 -7.527353000 -0.772770000 1.565914000  1 -5.084795000 -1.269044000 1.383997000  1 -4.802569000 1.425253000 -2.009927000  8 1.659572000 0.415031000 0.745720000  15 1.659571000 1.768440000 0.051074000  8 1.505260000 1.817859000 -1.572237000  1 0.609665000 1.475469000 -1.902245000  6 3.253803000 2.643177000 0.253257000  6 4.013152000 2.365139000 1.410427000  6 3.739928000 3.567022000 -0.697223000  6 5.237819000 3.019340000 1.623807000  6 4.967247000 4.216334000 -0.480909000  6 5.714284000 3.947569000 0.680399000  1 3.638326000 1.621194000 2.131299000  1 3.160549000 3.760108000 -1.612933000  1 5.826748000 2.800130000 2.528925000  1 5.345585000 4.934499000 -1.226110000  1 6.675173000 4.460225000 0.848089000  6 0.327308000 2.846598000 0.702291000  6 -0.077965000 4.020534000 0.029981000  6 -0.300992000 2.478959000 1.911395000  6 -1.098564000 4.821055000 0.568537000  6 -1.321934000 3.282897000 2.445875000  6 -1.719342000 4.454276000 1.776884000  1 0.396184000 4.300340000 -0.923921000  1 0.012387000 1.549386000 2.411797000  1 -1.417524000 5.732895000 0.038801000  1 -1.815947000 2.989259000 3.385796000  1 -2.522819000 5.081718000 2.194862000 |
| 66 bp86 2 optimized with GD3 (Figure 1) (Gaussian 16) 46 -0.037524000 -1.749886000 0.383348000  7 1.537005000 -1.042420000 1.699102000  6 1.399901000 -0.416508000 2.871351000  6 2.499912000 0.146549000 3.560460000  6 3.770413000 0.060946000 3.000172000  6 3.941857000 -0.600538000 1.754241000  6 2.777575000 -1.149586000 1.142088000  1 0.369556000 -0.332904000 3.252471000  1 2.329680000 0.659919000 4.517895000  1 4.642972000 0.507321000 3.503405000  6 5.210103000 -0.722161000 1.082417000  6 2.887579000 -1.826182000 -0.129346000  7 1.748058000 -2.341947000 -0.675324000  6 4.155954000 -1.926233000 -0.775393000  6 1.803863000 -2.963054000 -1.859405000  6 5.313608000 -1.355334000 -0.135373000  6 4.193703000 -2.589233000 -2.032264000  1 6.287875000 -1.435375000 -0.642889000  1 6.101241000 -0.287225000 1.561994000  6 3.018350000 -3.106800000 -2.572070000  1 0.838923000 -3.347792000 -2.235196000  1 3.015582000 -3.624601000 -3.542614000  1 5.152335000 -2.684336000 -2.567187000  6 -1.622270000 -0.886590000 1.180159000  8 -1.582787000 0.048087000 2.017989000  7 -2.827403000 -1.297882000 0.635138000  8 -2.747108000 -2.287082000 -0.364470000  6 -1.415133000 -2.579381000 -0.751169000  8 -1.310129000 -3.358155000 -1.677588000  6 -4.118148000 -0.719695000 0.721842000  6 -5.137354000 -1.152725000 -0.161723000  6 -6.400887000 -0.545258000 -0.123877000  6 -6.678083000 0.489492000 0.786193000  6 -5.668844000 0.900517000 1.674398000  6 -4.397944000 0.306212000 1.658405000  1 -7.179091000 -0.890629000 -0.823168000  1 -7.671108000 0.964578000 0.808366000  1 -5.868143000 1.702657000 2.403017000  1 -3.612627000 0.636371000 2.346627000  1 -4.924238000 -1.950417000 -0.882847000  8 2.012904000 1.067968000 -0.356496000  15 0.760619000 1.892079000 -0.103608000  8 0.142532000 1.959346000 1.404082000  1 -0.487936000 1.196552000 1.634036000  6 1.045436000 3.662272000 -0.435849000  6 2.122125000 4.010552000 -1.277843000  6 0.215924000 4.669094000 0.102072000  6 2.358539000 5.359240000 -1.592138000  6 0.456321000 6.016241000 -0.214852000  6 1.524220000 6.361533000 -1.064152000  1 2.771321000 3.210174000 -1.667223000  1 -0.604671000 4.390881000 0.781251000  1 3.200116000 5.631320000 -2.249170000  1 -0.189366000 6.803033000 0.206988000  1 1.709841000 7.419119000 -1.311493000  6 -0.626590000 1.314698000 -1.144912000  6 -0.337260000 0.420219000 -2.197443000  6 -1.963934000 1.667485000 -0.865840000  6 -1.383032000 -0.133668000 -2.952831000  6 -3.005971000 1.111802000 -1.624285000  6 -2.716184000 0.203797000 -2.659002000  1 0.711545000 0.137994000 -2.378718000  1 -2.195948000 2.337715000 -0.023873000  1 -1.160536000 -0.861962000 -3.747635000  1 -4.051165000 1.356622000 -1.381091000  1 -3.538746000 -0.257237000 -3.227569000 |
| 52 bp86_(Benzene + palladacycle 1) adduct NO dispersion (Figure 2 a) ORCA 6.0 46 0.286900000 0.739317000 0.446341000  7 1.725628000 1.041220000 -1.166764000  6 1.448530000 1.426679000 -2.418006000  6 2.464319000 1.598280000 -3.391295000  6 3.792700000 1.361177000 -3.047372000  6 4.110615000 0.949741000 -1.722707000  6 3.022515000 0.803431000 -0.808846000  1 0.373058000 1.596706000 -2.620599000  1 2.187392000 1.916753000 -4.407330000  1 4.600540000 1.487195000 -3.786194000  6 5.449710000 0.676598000 -1.263828000  6 3.277276000 0.386863000 0.553182000  7 2.203050000 0.262861000 1.386999000  6 4.615048000 0.121679000 0.978047000  6 2.395288000 -0.123033000 2.653848000  6 5.691970000 0.278635000 0.032674000  6 4.795833000 -0.286946000 2.329088000  1 6.720654000 0.072631000 0.368648000  1 6.282717000 0.792233000 -1.975325000  6 3.686801000 -0.408714000 3.162632000  1 1.475536000 -0.199143000 3.264217000  1 3.794321000 -0.723300000 4.211355000  1 5.810399000 -0.502978000 2.700887000  6 -1.413153000 1.187089000 -0.480715000  8 -1.507130000 1.536316000 -1.662671000  7 -2.532816000 1.071639000 0.345067000  8 -2.295129000 0.667391000 1.676817000  6 -0.936702000 0.448454000 1.959725000  8 -0.693868000 0.109451000 3.086915000  6 -3.902345000 1.323449000 0.086424000  6 -4.854654000 1.172092000 1.125342000  6 -6.212151000 1.419858000 0.871682000  6 -6.648691000 1.818294000 -0.402909000  6 -5.699768000 1.966303000 -1.429572000  6 -4.336729000 1.724506000 -1.202861000  1 -6.937461000 1.297255000 1.692203000  1 -7.715880000 2.010822000 -0.594483000  1 -6.020724000 2.277239000 -2.436958000  1 -3.598249000 1.841050000 -2.004277000  1 -4.522914000 0.861825000 2.123588000  6 -0.315699000 -3.498160000 -0.940527000  6 -1.511512000 -3.195964000 -0.263896000  6 -0.087616000 -4.799865000 -1.422881000  6 -2.479955000 -4.197614000 -0.069783000  6 -1.056495000 -5.801095000 -1.228276000  6 -2.252564000 -5.499545000 -0.551638000  1 -1.684415000 -2.174950000 0.111699000  1 0.849026000 -5.036060000 -1.953789000  1 -3.416673000 -3.960407000 0.459533000  1 -0.879163000 -6.821144000 -1.606105000  1 -3.011837000 -6.283763000 -0.399667000  1 0.438879000 -2.708996000 -1.089874000 |
| 52 bp86_(Benzene + palladacycle 1) adduct D3 (Figure 2 b) ORCA 6.0 46 0.132350000 0.464350000 -0.994791000  7 1.465608000 -1.245210000 -0.892104000  6 1.125092000 -2.527976000 -1.052226000  6 2.027144000 -3.583519000 -0.774678000  6 3.306648000 -3.289915000 -0.312388000  6 3.684511000 -1.933060000 -0.119320000  6 2.711522000 -0.935970000 -0.429977000  1 0.082217000 -2.693371000 -1.379250000  1 1.700179000 -4.622843000 -0.922954000  1 4.026049000 -4.092155000 -0.084678000  6 4.969613000 -1.523514000 0.386266000  6 3.020063000 0.459658000 -0.213427000  7 2.043664000 1.372189000 -0.487428000  6 4.296152000 0.833928000 0.305109000  6 2.275611000 2.670096000 -0.264282000  6 5.263991000 -0.194160000 0.589975000  6 4.525150000 2.219836000 0.525085000  1 6.247680000 0.104791000 0.983910000  1 5.714945000 -2.300489000 0.616490000  6 3.514335000 3.134051000 0.241093000  1 1.424709000 3.341504000 -0.478310000  1 3.657693000 4.211404000 0.407965000  1 5.495895000 2.553542000 0.924094000  6 -1.604119000 -0.469766000 -1.166299000  8 -1.762620000 -1.678924000 -1.356277000  7 -2.700377000 0.374138000 -0.912905000  8 -2.410641000 1.746844000 -0.775520000  6 -1.033321000 2.038877000 -0.833535000  8 -0.748505000 3.217211000 -0.729988000  6 -3.965730000 0.032249000 -0.386730000  6 -4.733077000 1.005546000 0.300971000  6 -5.970528000 0.655170000 0.860309000  6 -6.470719000 -0.652450000 0.747380000  6 -5.712764000 -1.610453000 0.052309000  6 -4.475129000 -1.284455000 -0.520820000  1 -6.550446000 1.425093000 1.393147000  1 -7.442872000 -0.920110000 1.188307000  1 -6.091652000 -2.638792000 -0.057937000  1 -3.887914000 -2.033880000 -1.062126000  1 -4.351840000 2.029764000 0.388703000  6 -0.395742000 -1.763855000 2.048153000  6 0.850886000 -1.172849000 2.319512000  6 -1.553061000 -0.968719000 1.987286000  6 0.941773000 0.216197000 2.518461000  6 -1.463126000 0.418535000 2.191741000  6 -0.215480000 1.012695000 2.451314000  1 1.759883000 -1.794249000 2.355261000  1 -2.526029000 -1.421865000 1.742989000  1 1.922114000 0.682339000 2.706351000  1 -2.367329000 1.040159000 2.107079000  1 -0.143312000 2.103720000 2.576866000  1 -0.464097000 -2.847020000 1.863831000 |
| 52 bp86_(Benzene + palladacycle 1) adduct NO dispersion (Figure 2 c) ORCA 6.0 46 -0.218788000 0.399127000 -0.591476000  7 1.166201000 -1.246157000 -0.826073000  6 0.852002000 -2.543824000 -0.875236000  6 1.845140000 -3.551219000 -0.876276000  6 3.188635000 -3.192018000 -0.811980000  6 3.541299000 -1.818370000 -0.743683000  6 2.474109000 -0.872413000 -0.758922000  1 -0.234215000 -2.753637000 -0.889876000  1 1.540782000 -4.606632000 -0.922383000  1 3.980509000 -3.957043000 -0.802857000  6 4.894797000 -1.339888000 -0.634335000  6 2.762455000 0.539138000 -0.664533000  7 1.705563000 1.398016000 -0.653150000  6 4.112358000 0.983339000 -0.548020000  6 1.922335000 2.710144000 -0.523290000  6 5.169239000 0.005505000 -0.538835000  6 4.323761000 2.382282000 -0.422334000  1 6.208411000 0.356939000 -0.448130000  1 5.712074000 -2.077113000 -0.621036000  6 3.228549000 3.241956000 -0.408562000  1 1.009393000 3.332477000 -0.492249000  1 3.360150000 4.328515000 -0.304960000  1 5.349957000 2.770152000 -0.328347000  6 -1.931628000 -0.585812000 -0.500815000  8 -2.058542000 -1.814665000 -0.569842000  7 -3.029053000 0.251560000 -0.301413000  8 -2.765126000 1.633265000 -0.224995000  6 -1.400740000 1.947254000 -0.358102000  8 -1.130504000 3.131468000 -0.280986000  6 -4.398941000 -0.050881000 -0.143622000  6 -5.328634000 0.995170000 0.070641000  6 -6.689245000 0.698475000 0.230527000  6 -7.150186000 -0.626762000 0.181373000  6 -6.222641000 -1.660050000 -0.032639000  6 -4.857163000 -1.390616000 -0.195732000  1 -7.397843000 1.524917000 0.396010000  1 -8.219840000 -0.851997000 0.307433000  1 -6.563510000 -2.706320000 -0.076110000  1 -4.133566000 -2.195209000 -0.363480000  1 -4.974840000 2.031574000 0.108624000  6 1.812319000 -1.712268000 2.412262000  6 2.799762000 -0.713022000 2.476569000  6 0.453422000 -1.358516000 2.404403000  6 2.425888000 0.641049000 2.545433000  6 0.079883000 -0.003544000 2.459731000  6 1.067514000 0.995480000 2.539406000  1 3.865260000 -0.990135000 2.469653000  1 -0.322955000 -2.134221000 2.326821000  1 3.198796000 1.423782000 2.589684000  1 -0.983830000 0.274419000 2.452967000  1 0.769067000 2.054114000 2.569791000  1 2.105548000 -2.771841000 2.355259000 |
| 52 bp86_(Benzene + palladacycle 1) adduct D4 (Figure 2 d) ORCA 6.0 46 -0.289251000 0.440540000 -0.455892000  7 1.032077000 -1.266565000 -0.589273000  6 0.670247000 -2.540262000 -0.763287000  6 1.610509000 -3.595937000 -0.717544000  6 2.950471000 -3.311518000 -0.472397000  6 3.352277000 -1.965180000 -0.271634000  6 2.337936000 -0.967202000 -0.348391000  1 -0.414355000 -2.694793000 -0.916879000  1 1.267577000 -4.629210000 -0.871369000  1 3.701145000 -4.115754000 -0.423439000  6 4.702588000 -1.565389000 0.022556000  6 2.677486000 0.419579000 -0.141263000  7 1.669455000 1.331355000 -0.216010000  6 4.022220000 0.785711000 0.154382000  6 1.932203000 2.622993000 -0.000518000  6 5.024199000 -0.243846000 0.230414000  6 4.283225000 2.163793000 0.374370000  1 6.060437000 0.047593000 0.460919000  1 5.478707000 -2.343661000 0.084349000  6 3.237803000 3.079252000 0.297766000  1 1.055675000 3.294088000 -0.059404000  1 3.407892000 4.152286000 0.466722000  1 5.308094000 2.492052000 0.607931000  6 -2.046454000 -0.454407000 -0.604249000  8 -2.222944000 -1.674308000 -0.712618000  7 -3.117450000 0.435833000 -0.533082000  8 -2.796752000 1.800070000 -0.392286000  6 -1.413504000 2.042861000 -0.330397000  8 -1.096334000 3.209545000 -0.191671000  6 -4.509467000 0.205876000 -0.577974000  6 -5.404196000 1.299484000 -0.489398000  6 -6.786976000 1.075467000 -0.534258000  6 -7.304632000 -0.222828000 -0.666502000  6 -6.411296000 -1.303559000 -0.753949000  6 -5.024585000 -1.107222000 -0.711554000  1 -7.467980000 1.938145000 -0.464621000  1 -8.391674000 -0.390862000 -0.701327000  1 -6.796589000 -2.330060000 -0.858343000  1 -4.327791000 -1.949348000 -0.780007000  1 -5.006593000 2.315567000 -0.387321000  6 1.351771000 -2.000659000 2.707924000  6 2.459219000 -1.177459000 2.978421000  6 0.077796000 -1.433720000 2.542852000  6 2.288161000 0.213499000 3.094814000  6 -0.092156000 -0.041761000 2.652205000  6 1.012764000 0.779914000 2.937735000  1 3.458990000 -1.621210000 3.103056000  1 -0.787794000 -2.071222000 2.307664000  1 3.154147000 0.857990000 3.310901000  1 -1.091412000 0.400325000 2.529817000  1 0.875627000 1.868506000 3.023970000  1 1.484539000 -3.090064000 2.620101000  78 bp86_(Benzene + palladacycle 1) adduct D3BJ (Figure 2 d) Gaussian 16 (used for RMSD) 46 -0.218788000 0.399127000 -0.591476000  7 1.166201000 -1.246157000 -0.826073000  6 0.852002000 -2.543824000 -0.875236000  6 1.845140000 -3.551219000 -0.876276000  6 3.188635000 -3.192018000 -0.811980000  6 3.541299000 -1.818370000 -0.743683000  6 2.474109000 -0.872413000 -0.758922000  1 -0.234215000 -2.753637000 -0.889876000  1 1.540782000 -4.606632000 -0.922383000  1 3.980509000 -3.957043000 -0.802857000  6 4.894797000 -1.339888000 -0.634335000  6 2.762455000 0.539138000 -0.664533000  7 1.705563000 1.398016000 -0.653150000  6 4.112358000 0.983339000 -0.548020000  6 1.922335000 2.710144000 -0.523290000  6 5.169239000 0.005505000 -0.538835000  6 4.323761000 2.382282000 -0.422334000  1 6.208411000 0.356939000 -0.448130000  1 5.712074000 -2.077113000 -0.621036000  6 3.228549000 3.241956000 -0.408562000  1 1.009393000 3.332477000 -0.492249000  1 3.360150000 4.328515000 -0.304960000  1 5.349957000 2.770152000 -0.328347000  6 -1.931628000 -0.585812000 -0.500815000  8 -2.058542000 -1.814665000 -0.569842000  7 -3.029053000 0.251560000 -0.301413000  8 -2.765126000 1.633265000 -0.224995000  6 -1.400740000 1.947254000 -0.358102000  8 -1.130504000 3.131468000 -0.280986000  6 -4.398941000 -0.050881000 -0.143622000  6 -5.328634000 0.995170000 0.070641000  6 -6.689245000 0.698475000 0.230527000  6 -7.150186000 -0.626762000 0.181373000  6 -6.222641000 -1.660050000 -0.032639000  6 -4.857163000 -1.390616000 -0.195732000  1 -7.397843000 1.524917000 0.396010000  1 -8.219840000 -0.851997000 0.307433000  1 -6.563510000 -2.706320000 -0.076110000  1 -4.133566000 -2.195209000 -0.363480000  1 -4.974840000 2.031574000 0.108624000  6 1.812319000 -1.712268000 2.412262000  6 2.799762000 -0.713022000 2.476569000  6 0.453422000 -1.358516000 2.404403000  6 2.425888000 0.641049000 2.545433000  6 0.079883000 -0.003544000 2.459731000  6 1.067514000 0.995480000 2.539406000  1 3.865260000 -0.990135000 2.469653000  1 -0.322955000 -2.134221000 2.326821000  1 3.198796000 1.423782000 2.589684000  1 -0.983830000 0.274419000 2.452967000  1 0.769067000 2.054114000 2.569791000  1 2.105548000 -2.771841000 2.355259000 |
| 78 bp86_4-methyl phenanthroline system with D4 (Figure 3 a) ORCA 6.0 46 -0.051820000 -2.172410000 0.535910000  7 1.562650000 -1.275040000 1.634680000  6 1.472250000 -0.502070000 2.717600000  6 2.513110000 0.339860000 3.166510000  6 3.724470000 0.358630000 2.449940000  6 3.864760000 -0.528570000 1.336970000  6 2.744630000 -1.323050000 0.962890000  1 0.489610000 -0.495260000 3.214570000  6 5.047510000 -0.629370000 0.523540000  6 2.808580000 -2.188870000 -0.190190000  7 1.682310000 -2.884740000 -0.511350000  6 3.992640000 -2.268630000 -0.973140000  6 1.690750000 -3.672490000 -1.585410000  6 5.113320000 -1.460110000 -0.572520000  6 3.997180000 -3.126150000 -2.120340000  1 6.041980000 -1.499890000 -1.158330000  1 5.925350000 -0.022160000 0.782820000  6 2.822840000 -3.841060000 -2.423630000  1 0.729530000 -4.169260000 -1.808260000  6 -1.560890000 -1.139300000 1.249040000  8 -1.498050000 -0.252130000 2.130200000  7 -2.753340000 -1.354070000 0.572400000  8 -2.744190000 -2.374990000 -0.392170000  6 -1.457550000 -2.932130000 -0.597180000  8 -1.406510000 -3.802350000 -1.442240000  6 -3.867350000 -0.492090000 0.440970000  6 -4.751270000 -0.657380000 -0.651980000  6 -5.827230000 0.225240000 -0.819120000  6 -6.048100000 1.273400000 0.088500000  6 -5.179550000 1.419390000 1.182540000  6 -4.099340000 0.546830000 1.375210000  1 -6.503080000 0.084130000 -1.676860000  1 -6.894640000 1.962680000 -0.049540000  1 -5.345530000 2.224530000 1.915210000  1 -3.429210000 0.660150000 2.232850000  1 -4.585300000 -1.478280000 -1.359020000  8 2.196570000 2.460750000 1.050280000  15 0.702080000 2.442210000 0.785510000  8 -0.278730000 2.037960000 2.015420000  1 -0.670490000 1.102410000 2.013690000  6 0.067440000 4.089610000 0.345260000  6 0.962930000 5.016580000 -0.223610000  6 -1.283830000 4.441000000 0.538270000  6 0.504500000 6.283960000 -0.614070000  6 -1.738470000 5.708780000 0.144280000  6 -0.846500000 6.628540000 -0.434380000  1 2.019660000 4.731490000 -0.341370000  1 -1.968700000 3.723290000 1.014190000  1 1.204440000 7.009610000 -1.056830000  1 -2.793690000 5.984400000 0.295600000  1 -1.205800000 7.623080000 -0.741730000  6 0.282950000 1.314820000 -0.587240000  6 1.345090000 0.680840000 -1.259900000  6 -1.047060000 1.076510000 -0.989130000  6 1.076830000 -0.205820000 -2.313610000  6 -1.310090000 0.193310000 -2.045570000  6 -0.249730000 -0.455100000 -2.702050000  1 2.374140000 0.894570000 -0.934270000  1 -1.884620000 1.569910000 -0.472900000  1 1.907800000 -0.712890000 -2.828360000  1 -2.350570000 -0.003290000 -2.343760000  1 -0.462770000 -1.164380000 -3.515880000  6 2.706930000 -4.767830000 -3.609270000  1 3.433350000 -5.605220000 -3.549980000  1 1.693110000 -5.207860000 -3.664120000  1 2.895580000 -4.239570000 -4.567120000  6 5.237330000 -3.232300000 -2.968450000  1 6.103530000 -3.581520000 -2.368080000  1 5.110850000 -3.932370000 -3.812560000  1 5.520260000 -2.244450000 -3.389350000  6 4.768230000 1.379710000 2.788980000  1 4.880550000 1.509350000 3.882690000  1 5.763580000 1.157520000 2.364940000  1 4.419250000 2.350380000 2.371790000  6 2.240830000 1.295980000 4.292600000  1 1.263170000 1.091460000 4.767460000  1 3.026430000 1.261200000 5.075360000  1 2.209650000 2.330110000 3.889570000 |
| 93 bp86_ Toluene + 4-methyl phenanthroline system with D3BJ (Figure 3 b) Gaussian 16 46 0.138994000 -1.294013000 -0.256125000  7 0.725769000 0.260077000 1.110490000  6 0.097643000 0.647782000 2.216039000  6 0.560872000 1.686916000 3.066532000  6 1.739295000 2.363997000 2.714910000  6 2.432697000 1.945429000 1.530996000  6 1.892163000 0.877605000 0.766284000  1 -0.842929000 0.114528000 2.433045000  6 3.654542000 2.546997000 1.065178000  6 2.570889000 0.400612000 -0.410393000  7 2.008511000 -0.644739000 -1.074995000  6 3.786624000 1.002270000 -0.842971000  6 2.611848000 -1.128448000 -2.159151000  6 4.300441000 2.099997000 -0.068507000  6 4.429583000 0.478597000 -2.009411000  1 5.234290000 2.588229000 -0.381518000  1 4.086962000 3.384059000 1.631707000  6 3.828187000 -0.608876000 -2.672031000  1 2.098342000 -1.983518000 -2.635506000  6 -1.600802000 -1.821597000 0.504767000  8 -2.234027000 -1.193106000 1.391366000  7 -2.136279000 -2.962164000 -0.059340000  8 -1.388575000 -3.559672000 -1.092878000  6 -0.220940000 -2.828604000 -1.428203000  8 0.414102000 -3.290590000 -2.357112000  6 -3.379804000 -3.607606000 0.155192000  6 -3.773670000 -4.662005000 -0.702946000  6 -5.003189000 -5.304614000 -0.498285000  6 -5.853116000 -4.917627000 0.550890000  6 -5.453095000 -3.873324000 1.401892000  6 -4.228194000 -3.215785000 1.219595000  1 -5.296016000 -6.122653000 -1.175300000  1 -6.817361000 -5.425895000 0.705213000  1 -6.104539000 -3.556857000 2.231808000  1 -3.919075000 -2.399271000 1.880550000  1 -3.112088000 -4.968704000 -1.521516000  8 -1.497275000 2.957539000 1.471825000  15 -2.224327000 2.359565000 0.280059000  8 -3.186624000 1.074244000 0.496293000  1 -2.750804000 0.245316000 0.889759000  6 -3.398198000 3.540391000 -0.458484000  6 -3.347445000 4.877789000 -0.018669000  6 -4.321568000 3.157501000 -1.453437000  6 -4.212761000 5.832404000 -0.578214000  6 -5.184403000 4.114681000 -2.009265000  6 -5.129012000 5.451996000 -1.574237000  1 -2.627345000 5.145376000 0.770616000  1 -4.368409000 2.108779000 -1.784692000  1 -4.175254000 6.877887000 -0.233130000  1 -5.908088000 3.816718000 -2.784340000  1 -5.807228000 6.200794000 -2.013420000  6 -1.061850000 1.913078000 -1.060985000  6 0.104316000 2.691065000 -1.215222000  6 -1.324124000 0.856730000 -1.959692000  6 0.984712000 2.434772000 -2.278248000  6 -0.438978000 0.603302000 -3.023123000  6 0.707805000 1.397190000 -3.187860000  1 0.313734000 3.488898000 -0.485840000  1 -2.228061000 0.242927000 -1.831055000  1 1.894827000 3.043387000 -2.395077000  1 -0.638162000 -0.232434000 -3.711113000  1 1.401157000 1.195111000 -4.018949000  6 -0.307740000 2.069797000 4.234163000  1 -0.927900000 2.943652000 3.942172000  1 -1.005916000 1.248661000 4.487666000  1 0.272628000 2.324105000 5.142866000  6 2.268146000 3.524982000 3.515557000  1 1.661348000 3.720589000 4.416875000  1 3.316581000 3.354237000 3.839245000  1 2.264383000 4.453881000 2.906089000  6 4.421134000 -1.250853000 -3.903697000  1 4.525438000 -0.526530000 -4.739059000  1 5.431379000 -1.667342000 -3.705484000  1 3.780452000 -2.081365000 -4.257859000  6 5.723809000 1.086031000 -2.485538000  1 6.513541000 0.997336000 -1.709101000  1 6.101911000 0.604368000 -3.404575000  1 5.605407000 2.169929000 -2.696367000  6 4.471195000 -0.819850000 2.188631000  6 3.280055000 -1.145289000 2.859787000  6 4.840954000 -1.521866000 1.029012000  1 2.989094000 -0.582272000 3.761661000  1 5.771966000 -1.267101000 0.499089000  6 2.434116000 -2.169874000 2.387662000  6 4.008808000 -2.546996000 0.547128000  1 4.280330000 -3.095837000 -0.368175000  6 2.814177000 -2.860022000 1.214324000  1 2.157923000 -3.649871000 0.815955000  1 5.108576000 -0.006098000 2.568381000  6 1.147249000 -2.519085000 3.093594000  1 0.926269000 -1.811611000 3.916767000  1 0.296517000 -2.494455000 2.380326000  1 1.186271000 -3.542894000 3.522714000 |
| 66 Intermediate $\mathbf{2}_{\boldsymbol{conf}\mathbf{1}}$- used for benchmarks in Table 2 and 3 46 0.520994000 1.315529000 -0.726513000  7 -0.507751000 2.473700000 0.763967000  6 0.004068000 2.905382000 1.921125000  6 -0.811036000 3.475290000 2.927477000  6 -2.182904000 3.590126000 2.715197000  6 -2.744702000 3.135527000 1.491527000  6 -1.848579000 2.574637000 0.533993000  1 1.097608000 2.771419000 2.021010000  1 -0.349422000 3.817922000 3.865152000  1 -2.840230000 4.026221000 3.484221000  6 -4.148476000 3.203162000 1.176992000  6 -2.354876000 2.074105000 -0.720514000  7 -1.462463000 1.510573000 -1.582253000  6 -3.747845000 2.157367000 -1.005845000  6 -1.896875000 1.019057000 -2.745375000  6 -4.628592000 2.736375000 -0.025492000  6 -4.187118000 1.629023000 -2.248589000  1 -5.704588000 2.788529000 -0.253731000  1 -4.833572000 3.635701000 1.922669000  6 -3.260289000 1.064367000 -3.118682000  1 -1.132672000 0.547155000 -3.383727000  1 -3.568629000 0.636159000 -4.083235000  1 -5.257781000 1.661869000 -2.504932000  6 2.352299000 1.307919000 0.028265000  8 2.733907000 1.946165000 1.012815000  7 3.244342000 0.517375000 -0.710873000  8 2.685094000 -0.203653000 -1.788440000  6 1.326821000 0.026857000 -1.949349000  8 0.779666000 -0.688672000 -2.793111000  6 4.534727000 0.056229000 -0.369217000  6 5.111145000 -1.022829000 -1.082174000  6 6.379923000 -1.499943000 -0.721528000  6 7.094514000 -0.920840000 0.340109000  6 6.521109000 0.157467000 1.035773000  6 5.256103000 0.655862000 0.692523000  1 6.812434000 -2.341167000 -1.286145000  1 8.089527000 -1.300876000 0.617961000  1 7.068491000 0.631642000 1.865979000  1 4.807167000 1.493561000 1.236934000  1 4.558673000 -1.480850000 -1.910934000  8 -2.565828000 -0.481868000 0.305409000  15 -1.671734000 -1.645614000 -0.085793000  8 -1.340558000 -1.906650000 -1.660911000  1 -0.579789000 -1.379906000 -2.058442000  6 -0.055479000 -1.545074000 0.757597000  6 1.084076000 -2.236680000 0.295347000  6 0.031471000 -0.735692000 1.910788000  6 2.307420000 -2.093323000 0.967341000  6 1.255524000 -0.602081000 2.581693000  6 2.395695000 -1.272566000 2.105189000  1 1.022446000 -2.855434000 -0.612649000  1 -0.868232000 -0.196942000 2.245277000  1 3.207039000 -2.600563000 0.587329000  1 1.330246000 0.049741000 3.465649000  1 3.365624000 -1.140085000 2.608012000  6 -2.430597000 -3.239518000 0.358092000  6 -3.458978000 -3.229569000 1.322228000  6 -2.002757000 -4.459372000 -0.205494000  6 -4.048260000 -4.437000000 1.730367000  6 -2.594894000 -5.663918000 0.205637000  6 -3.614354000 -5.653983000 1.175284000  1 -3.790215000 -2.261883000 1.730480000  1 -1.217553000 -4.456959000 -0.976567000  1 -4.853022000 -4.429572000 2.482519000  1 -2.263682000 -6.616997000 -0.236184000  1 -4.076437000 -6.601097000 1.496171000 |
| 66 Intermediate $\mathbf{2}_{\boldsymbol{conf}\mathbf{2}}$ used for benchmarks in Table 2 and 3 46 1.081882000 -0.746158000 0.322049000  7 0.117795000 -2.215025000 -0.920843000  6 0.563109000 -2.651011000 -2.104006000  6 -0.201598000 -3.528273000 -2.910328000  6 -1.442601000 -3.971536000 -2.459529000  6 -1.928808000 -3.527476000 -1.199741000  6 -1.105515000 -2.618654000 -0.469426000  1 1.575357000 -2.296966000 -2.375596000  1 0.203254000 -3.859123000 -3.877990000  1 -2.049500000 -4.666685000 -3.061260000  6 -3.182378000 -3.945940000 -0.628552000  6 -1.551087000 -2.109735000 0.805318000  7 -0.742796000 -1.215848000 1.444735000  6 -2.790854000 -2.554999000 1.353118000  6 -1.105002000 -0.755049000 2.646362000  6 -3.593603000 -3.482090000 0.600407000  6 -3.159131000 -2.039740000 2.623974000  1 -4.553955000 -3.809296000 1.027752000  1 -3.809455000 -4.649611000 -1.197980000  6 -2.307869000 -1.150859000 3.274414000  1 -0.413577000 -0.037749000 3.114924000  1 -2.560492000 -0.737731000 4.261611000  1 -4.112090000 -2.350232000 3.079874000  6 2.894507000 -0.757973000 -0.470629000  8 3.252674000 -1.417865000 -1.449965000  7 3.805002000 0.025767000 0.250956000  8 3.259573000 0.812525000 1.288388000  6 1.905905000 0.608377000 1.456213000  8 1.366342000 1.381399000 2.261935000  6 5.193443000 0.222117000 0.088575000  6 5.884710000 1.114662000 0.943589000  6 7.265726000 1.302175000 0.787459000  6 7.980087000 0.616883000 -0.208953000  6 7.287222000 -0.266389000 -1.054692000  6 5.907061000 -0.472410000 -0.919905000  1 7.787024000 2.000540000 1.461387000  1 9.063961000 0.769912000 -0.326027000  1 7.828130000 -0.813058000 -1.843617000  1 5.365186000 -1.157509000 -1.581232000  1 5.334300000 1.654695000 1.723001000  8 -0.269152000 1.179549000 -0.964662000  15 -1.331216000 1.805528000 -0.072484000  8 -1.028550000 2.016447000 1.505450000  1 -0.070786000 1.780017000 1.801054000  6 -2.864441000 0.810776000 -0.093402000  6 -3.080417000 -0.038501000 -1.200119000  6 -3.835922000 0.905331000 0.921698000  6 -4.272097000 -0.772352000 -1.297416000  6 -5.024591000 0.162715000 0.822817000  6 -5.246408000 -0.669287000 -0.288416000  1 -2.297772000 -0.113754000 -1.971226000  1 -3.651869000 1.553897000 1.791738000  1 -4.439724000 -1.434867000 -2.160576000  1 -5.784423000 0.235719000 1.617037000  1 -6.181870000 -1.245419000 -0.366538000  6 -1.818014000 3.462512000 -0.651371000  6 -2.233575000 4.466171000 0.247622000  6 -1.782059000 3.724777000 -2.036997000  6 -2.625839000 5.722549000 -0.241937000  6 -2.175873000 4.982226000 -2.520501000  6 -2.601547000 5.979515000 -1.624405000  1 -2.228685000 4.258991000 1.328575000  1 -1.426447000 2.941635000 -2.724726000  1 -2.945568000 6.509292000 0.459548000  1 -2.143298000 5.189492000 -3.601736000  1 -2.908378000 6.966383000 -2.005549000 |
| 66 Intermediate $\mathbf{2}_{\boldsymbol{conf}\boldsymbol{3}}$ used for benchmarks in Table 2 and 3 46 -2.150015000 1.066351000 0.047499000  7 -1.971924000 -0.950075000 0.821554000  6 -0.907524000 -1.506402000 1.406350000  6 -0.873649000 -2.870578000 1.787719000  6 -1.994647000 -3.662542000 1.570883000  6 -3.156078000 -3.088274000 0.979815000  6 -3.089907000 -1.710669000 0.617073000  1 -0.036661000 -0.854753000 1.568219000  1 0.073282000 -3.247344000 2.200904000  1 -1.998940000 -4.728961000 1.847594000  6 -4.373884000 -3.815643000 0.729538000  6 -4.237280000 -1.067860000 0.023713000  7 -4.126869000 0.256561000 -0.292519000  6 -5.435079000 -1.811025000 -0.202996000  6 -5.169773000 0.896517000 -0.832897000  6 -5.469600000 -3.203512000 0.161875000  6 -6.530631000 -1.111925000 -0.779861000  1 -6.395159000 -3.771667000 -0.019938000  1 -4.412830000 -4.880727000 1.006802000  6 -6.396467000 0.238433000 -1.091647000  1 -4.996818000 1.964762000 -1.059084000  1 -7.227009000 0.805455000 -1.536816000  1 -7.474191000 -1.646799000 -0.972576000  6 -0.297117000 1.725930000 0.232508000  8 0.700425000 1.035206000 0.548631000  7 -0.149793000 3.065717000 -0.060507000  8 -1.311390000 3.750805000 -0.464225000  6 -2.441529000 2.912645000 -0.563425000  8 -3.455556000 3.456568000 -0.953463000  6 1.008421000 3.883799000 -0.096915000  6 0.900630000 5.209391000 -0.582005000  6 2.033834000 6.034100000 -0.618347000  6 3.280308000 5.563037000 -0.174495000  6 3.378764000 4.247080000 0.307114000  6 2.262345000 3.399782000 0.351334000  1 1.932605000 7.062544000 -0.999558000  1 4.166449000 6.215836000 -0.202003000  1 4.343209000 3.854118000 0.661161000  1 2.353921000 2.373630000 0.723509000  1 -0.071799000 5.582049000 -0.924322000  8 2.084095000 -2.288104000 1.703382000  15 2.524212000 -1.785196000 0.337961000  8 1.398724000 -1.155427000 -0.656727000  1 1.070477000 -0.276064000 -0.259530000  6 3.224404000 -3.094333000 -0.711527000  6 3.146665000 -3.054390000 -2.119321000  6 3.869917000 -4.171670000 -0.069480000  6 3.729541000 -4.081864000 -2.878997000  6 4.452774000 -5.194439000 -0.834007000  6 4.386017000 -5.148011000 -2.238331000  1 2.613010000 -2.225595000 -2.608521000  1 3.896368000 -4.199006000 1.031314000  1 3.666999000 -4.053795000 -3.978370000  1 4.957953000 -6.035340000 -0.333090000  1 4.843549000 -5.951256000 -2.837428000  6 3.783852000 -0.474004000 0.510855000  6 4.327749000 0.185378000 -0.611108000  6 4.195979000 -0.116697000 1.809594000  6 5.297029000 1.183552000 -0.432338000  6 5.161938000 0.888049000 1.984619000  6 5.719263000 1.529995000 0.864828000  1 3.991491000 -0.082404000 -1.624861000  1 3.741854000 -0.638743000 2.666169000  1 5.721349000 1.700371000 -1.307090000  1 5.484352000 1.169729000 2.999407000  1 6.486174000 2.308855000 1.002541000 |
| 66 Intermediate $\mathbf{2}_{\boldsymbol{conf}\mathbf{4}}$ used for benchmarks in Table 2 and 3 46 -0.683068000 -1.440008000 -0.175306000  7 0.838652000 -1.126969000 1.362343000  6 0.677417000 -0.586779000 2.574978000  6 1.747735000 -0.452301000 3.488122000  6 3.020286000 -0.880178000 3.119970000  6 3.215323000 -1.470141000 1.843380000  6 2.073731000 -1.577051000 0.994482000  1 -0.341740000 -0.241971000 2.811219000  1 1.561383000 0.003029000 4.471499000  1 3.876735000 -0.769836000 3.803144000  6 4.485169000 -1.953308000 1.369328000  6 2.200955000 -2.192543000 -0.304166000  7 1.068783000 -2.325679000 -1.054863000  6 3.475322000 -2.653398000 -0.752566000  6 1.142670000 -2.881158000 -2.269498000  6 4.611873000 -2.518648000 0.120794000  6 3.534765000 -3.230125000 -2.050185000  1 5.590705000 -2.879019000 -0.232239000  1 5.361067000 -1.855027000 2.028985000  6 2.369900000 -3.338348000 -2.806544000  1 0.177850000 -2.978888000 -2.799157000  1 2.383450000 -3.785693000 -3.811205000  1 4.499524000 -3.591771000 -2.440037000  6 -2.255303000 -0.610676000 0.662268000  8 -2.211174000 0.287504000 1.548485000  7 -3.459747000 -1.025674000 0.144079000  8 -3.393127000 -1.944899000 -0.918384000  6 -2.062001000 -2.210362000 -1.334801000  8 -1.962340000 -2.927625000 -2.309448000  6 -4.780729000 -0.585788000 0.405909000  6 -5.829068000 -0.973298000 -0.461621000  6 -7.139116000 -0.543369000 -0.205804000  6 -7.427799000 0.268478000 0.903597000  6 -6.381725000 0.645601000 1.763098000  6 -5.064063000 0.226652000 1.530385000  1 -7.944692000 -0.851938000 -0.890727000  1 -8.458532000 0.602844000 1.097982000  1 -6.589123000 1.279444000 2.639881000  1 -4.248358000 0.526181000 2.197039000  1 -5.605743000 -1.608062000 -1.327178000  8 -0.024651000 0.881094000 -1.267532000  15 0.441984000 1.886160000 -0.222350000  8 -0.282440000 1.928142000 1.221043000  1 -1.074882000 1.262720000 1.339033000  6 2.210193000 1.633628000 0.169333000  6 3.012931000 0.983324000 -0.792632000  6 2.786539000 2.104012000 1.364948000  6 4.388191000 0.824594000 -0.565410000  6 4.163145000 1.936191000 1.590916000  6 4.964842000 1.304390000 0.624269000  1 2.537515000 0.602726000 -1.710184000  1 2.149762000 2.591073000 2.119145000  1 5.013557000 0.316706000 -1.315821000  1 4.614146000 2.303031000 2.526611000  1 6.044766000 1.179410000 0.801116000  6 0.321258000 3.600721000 -0.825724000  6 0.517970000 3.843989000 -2.201257000  6 0.048101000 4.672052000 0.049347000  6 0.456804000 5.156593000 -2.694632000  6 -0.012552000 5.983187000 -0.450003000  6 0.195820000 6.226469000 -1.819370000  1 0.700346000 2.994869000 -2.878395000  1 -0.132469000 4.465377000 1.115211000  1 0.605326000 5.346731000 -3.769304000  1 -0.230939000 6.820401000 0.231700000  1 0.146276000 7.255712000 -2.208676000  93 palladacycle 1 + Toluene on O – side 46 -0.308810000 -1.760219000 -0.750022000  7 -1.555049000 -0.297325000 -1.729183000  6 -1.214587000 0.530678000 -2.714784000  6 -2.097743000 1.491457000 -3.279473000  6 -3.390477000 1.608588000 -2.743305000  6 -3.771197000 0.717346000 -1.685409000  6 -2.821434000 -0.233486000 -1.222017000  1 -0.171879000 0.446493000 -3.063231000  6 -5.060775000 0.738952000 -1.046331000  6 -3.164850000 -1.162221000 -0.174758000  7 -2.211835000 -2.060681000 0.202901000  6 -4.447312000 -1.119305000 0.443688000  6 -2.477349000 -2.916478000 1.189852000  6 -5.383203000 -0.132948000 -0.027222000  6 -4.722405000 -2.036818000 1.509054000  1 -6.379318000 -0.073847000 0.434045000  1 -5.808194000 1.473894000 -1.377833000  6 -3.714619000 -2.946217000 1.885501000  1 -1.655287000 -3.613629000 1.435958000  6 1.433076000 -1.395079000 -1.590005000  8 1.728336000 -0.359314000 -2.234291000  7 2.380610000 -2.377985000 -1.369057000  8 1.968554000 -3.473488000 -0.586005000  6 0.676258000 -3.289645000 -0.014529000  8 0.342825000 -4.156203000 0.768830000  6 3.779942000 -2.367345000 -1.591767000  6 4.577407000 -3.407271000 -1.055968000  6 5.967702000 -3.384668000 -1.237345000  6 6.584992000 -2.345190000 -1.951854000  6 5.786143000 -1.324516000 -2.494721000  6 4.393754000 -1.324178000 -2.328393000  1 6.573570000 -4.198486000 -0.808776000  1 7.677233000 -2.332368000 -2.088143000  1 6.249965000 -0.503743000 -3.064288000  1 3.772926000 -0.528010000 -2.751817000  1 4.100721000 -4.222738000 -0.499614000  8 -0.194854000 3.122671000 -1.833537000  15 0.540371000 2.680883000 -0.580346000  8 1.888412000 1.799075000 -0.758724000  1 1.771943000 0.988661000 -1.359593000  6 1.153018000 4.107867000 0.371924000  6 1.140176000 5.372759000 -0.247459000  6 1.608505000 3.974186000 1.698427000  6 1.597072000 6.499872000 0.455482000  6 2.060385000 5.103276000 2.398946000  6 2.057722000 6.365707000 1.777208000  1 0.761595000 5.451507000 -1.278752000  1 1.599731000 2.985114000 2.179503000  1 1.590417000 7.489463000 -0.028473000  1 2.411551000 5.000453000 3.437672000  1 2.411197000 7.250870000 2.329588000  6 -0.530578000 1.753209000 0.574215000  6 -1.862949000 2.190305000 0.737441000  6 -0.047973000 0.664429000 1.329548000  6 -2.697091000 1.563287000 1.674910000  6 -0.892610000 0.035276000 2.262251000  6 -2.208669000 0.488108000 2.442139000  1 -2.234202000 3.024330000 0.121468000  1 0.989540000 0.322554000 1.206161000  1 -3.735907000 1.905422000 1.801642000  1 -0.508424000 -0.818760000 2.838679000  1 -2.866035000 -0.008694000 3.172995000  6 -1.576075000 2.379532000 -4.376870000  1 -1.378603000 3.391663000 -3.968924000  1 -0.611486000 1.997696000 -4.761246000  1 -2.284070000 2.458768000 -5.226773000  6 -4.361822000 2.655344000 -3.222935000  1 -3.939078000 3.273576000 -4.033971000  1 -5.303370000 2.198851000 -3.595399000  1 -4.640966000 3.340211000 -2.393915000  6 -3.889297000 -3.952206000 2.998409000  1 -4.097303000 -3.458652000 3.971263000  1 -4.731315000 -4.647813000 2.797848000  1 -2.973469000 -4.561988000 3.120719000  6 -6.060574000 -1.998421000 2.200704000  1 -6.889448000 -2.157097000 1.478935000  1 -6.149193000 -2.767445000 2.988252000  1 -6.235541000 -1.008635000 2.674113000  6 2.332478000 -1.559372000 3.007298000  6 2.245116000 -0.449606000 3.860796000  6 3.010375000 -1.452135000 1.779488000  1 1.718819000 -0.539421000 4.825851000  1 3.096958000 -2.329441000 1.126524000  6 2.828347000 0.787784000 3.505561000  6 3.581168000 -0.226176000 1.404043000  1 4.097714000 -0.133541000 0.436325000  6 3.488267000 0.884268000 2.261844000  1 3.933701000 1.844262000 1.956146000  1 1.874107000 -2.519178000 3.293254000  6 2.727368000 1.972086000 4.439661000  1 2.993290000 1.690653000 5.479934000  1 3.400293000 2.794543000 4.126385000  1 1.691162000 2.375105000 4.471395000  93 palladacycle 1 +Toluene on N – side |

46 -1.063696000 -1.704354000 -0.368642000

7 -1.440916000 0.096431000 -1.497391000

6 -0.550726000 0.847512000 -2.144846000

6 -0.871114000 2.084517000 -2.769995000

6 -2.188162000 2.564176000 -2.672798000

6 -3.156048000 1.759148000 -1.985128000

6 -2.735747000 0.523104000 -1.421838000

1 0.481165000 0.456444000 -2.144399000

6 -4.533106000 2.141190000 -1.808920000

6 -3.672339000 -0.315282000 -0.714153000

7 -3.197099000 -1.479427000 -0.188688000

6 -5.028993000 0.087276000 -0.549663000

6 -4.020284000 -2.264833000 0.506208000

6 -5.427380000 1.345789000 -1.123630000

6 -5.908892000 -0.764048000 0.195021000

1 -6.467968000 1.682100000 -1.011644000

1 -4.880323000 3.095454000 -2.230220000

6 -5.388446000 -1.958569000 0.730633000

1 -3.553597000 -3.179235000 0.917603000

6 0.896421000 -1.810008000 -0.495055000

8 1.642051000 -0.923496000 -0.979739000

7 1.437968000 -2.933005000 0.090723000

8 0.533880000 -3.830835000 0.690509000

6 -0.809275000 -3.385475000 0.621507000

8 -1.615535000 -4.109641000 1.173379000

6 2.793982000 -3.299977000 0.283798000

6 3.108230000 -4.324762000 1.207132000

6 4.445165000 -4.699096000 1.404648000

6 5.479284000 -4.071356000 0.690851000

6 5.157010000 -3.059490000 -0.229373000

6 3.829337000 -2.662937000 -0.443130000

1 4.675196000 -5.497469000 2.127798000

1 6.527208000 -4.370956000 0.846561000

1 5.947906000 -2.560632000 -0.808684000

1 3.597097000 -1.866555000 -1.159425000

1 2.300160000 -4.821679000 1.757320000

8 0.972460000 2.828288000 -0.501394000

15 1.098377000 2.074163000 0.813888000

8 2.115722000 0.816652000 0.898967000

1 2.005089000 0.165941000 0.121886000

6 1.743088000 3.136017000 2.144555000

6 1.954887000 4.498189000 1.855060000

6 2.028690000 2.631365000 3.430283000

6 2.448121000 5.356424000 2.851874000

6 2.521109000 3.492881000 4.422550000

6 2.729572000 4.854802000 4.134527000

1 1.734354000 4.861014000 0.838818000

1 1.869166000 1.564653000 3.651571000

1 2.617180000 6.421260000 2.626258000

1 2.747122000 3.101012000 5.426771000

1 3.115909000 5.528070000 4.916168000

6 -0.518293000 1.451196000 1.393705000

6 -1.667144000 2.233460000 1.152468000

6 -0.633769000 0.230065000 2.091011000

6 -2.916627000 1.811365000 1.631747000

6 -1.889050000 -0.189707000 2.567671000

6 -3.026100000 0.603559000 2.346181000

1 -1.569672000 3.166291000 0.575587000

1 0.261935000 -0.384936000 2.263121000

1 -3.813537000 2.419318000 1.435236000

1 -1.977305000 -1.153733000 3.091579000

1 -4.009586000 0.267811000 2.710780000

6 0.234535000 2.844679000 -3.452167000

1 0.576347000 3.672318000 -2.797448000

1 1.110395000 2.190257000 -3.617940000

1 -0.078299000 3.259558000 -4.431162000

6 -2.597551000 3.898824000 -3.239806000

1 -1.754236000 4.421400000 -3.723961000

1 -3.409016000 3.790618000 -3.990570000

1 -2.986000000 4.564106000 -2.439583000

6 -6.218266000 -2.926347000 1.540386000

1 -6.638185000 -2.448668000 2.450702000

1 -7.074352000 -3.324692000 0.955881000

1 -5.605273000 -3.788603000 1.866244000

6 -7.346590000 -0.359802000 0.396459000

1 -7.860317000 -0.209973000 -0.576573000

1 -7.919030000 -1.113197000 0.965788000

1 -7.417233000 0.603327000 0.945766000

6 3.621308000 1.397808000 -2.005522000

6 4.610864000 1.255138000 -1.021141000

6 3.728309000 0.688159000 -3.213926000

1 4.498328000 1.789240000 -0.064927000

1 2.946740000 0.780743000 -3.985183000

6 5.727241000 0.414987000 -1.221361000

6 4.832933000 -0.151323000 -3.432886000

1 4.925189000 -0.711489000 -4.376988000

6 5.826316000 -0.279036000 -2.446093000

1 6.695950000 -0.932245000 -2.629485000

1 2.753125000 2.044358000 -1.799744000

6 6.757295000 0.238243000 -0.130583000

1 7.044209000 1.210406000 0.321046000

1 6.354383000 -0.394832000 0.689793000

1 7.677071000 -0.251673000 -0.508974000

108

## palladacycle 1 + Toluene on N – side

46 -1.067023000 1.317932000 -0.531069000

7 -0.902042000 -0.456550000 0.684232000

6 0.107460000 -0.817448000 1.474600000

6 0.093627000 -1.978773000 2.295329000

6 -1.020512000 -2.832452000 2.224660000

6 -2.118538000 -2.446352000 1.389746000

6 -2.022810000 -1.234527000 0.653120000

1 0.984801000 -0.150945000 1.467273000

6 -3.331285000 -3.211022000 1.268144000

6 -3.128317000 -0.782356000 -0.151882000

7 -2.984959000 0.403766000 -0.804074000

6 -4.323933000 -1.550006000 -0.245748000

6 -3.989188000 0.868302000 -1.546075000

6 -4.384444000 -2.786446000 0.486339000

6 -5.398004000 -1.048986000 -1.049700000

1 -5.293985000 -3.401274000 0.430576000

1 -3.423608000 -4.155877000 1.822292000

6 -5.222412000 0.184485000 -1.706309000

1 -3.799493000 1.845095000 -2.027649000

6 0.716757000 2.113418000 -0.295721000

8 1.707086000 1.547524000 0.236067000

7 0.836020000 3.393984000 -0.792451000

8 -0.303637000 3.924797000 -1.426198000

6 -1.379396000 3.003106000 -1.492925000

8 -2.358305000 3.414544000 -2.085532000

6 1.938651000 4.285076000 -0.815649000

6 1.794114000 5.549226000 -1.435861000

6 2.874774000 6.442492000 -1.458744000

6 4.104737000 6.101929000 -0.872010000

6 4.241423000 4.845679000 -0.257832000

6 3.176484000 3.934212000 -0.222289000

1 2.745673000 7.421965000 -1.945756000

1 4.948659000 6.808460000 -0.893244000

1 5.196997000 4.557737000 0.207943000

1 3.291425000 2.955233000 0.252762000

1 0.835620000 5.820254000 -1.893582000

8 2.310170000 -2.564292000 0.241480000

15 2.335525000 -1.812784000 -1.079782000

8 2.901665000 -0.293515000 -1.123429000

1 2.377727000 0.377908000 -0.561001000

6 3.428090000 -2.616530000 -2.292413000

6 3.802951000 -3.954137000 -2.053596000

6 3.880902000 -1.950091000 -3.450060000

6 4.621949000 -4.626467000 -2.975178000

6 4.699995000 -2.626593000 -4.367410000

6 5.068151000 -3.964269000 -4.132452000

1 3.452604000 -4.446062000 -1.132589000

1 3.600705000 -0.899547000 -3.620545000

1 4.918324000 -5.670650000 -2.787759000

1 5.058695000 -2.106601000 -5.269687000

1 5.711162000 -4.491750000 -4.854774000

6 0.669878000 -1.745314000 -1.827971000

6 -0.222700000 -2.805693000 -1.566163000

6 0.267249000 -0.676828000 -2.656394000

6 -1.500440000 -2.806604000 -2.146302000

6 -1.015835000 -0.680392000 -3.232084000

6 -1.894697000 -1.747707000 -2.984620000

1 0.091231000 -3.612289000 -0.885741000

1 0.957202000 0.158517000 -2.847354000

1 -2.201108000 -3.627998000 -1.928934000

1 -1.333388000 0.165644000 -3.860412000

1 -2.904089000 -1.741308000 -3.425164000

6 1.298431000 -2.245632000 3.151928000

1 2.018172000 -2.866040000 2.578576000

1 1.821293000 -1.302597000 3.394077000

1 1.046202000 -2.755899000 4.100916000

6 -1.092006000 -4.124069000 2.997390000

1 -0.145881000 -4.346150000 3.520732000

1 -1.904268000 -4.098719000 3.755684000

1 -1.308362000 -4.978355000 2.322373000

6 -6.287903000 0.815788000 -2.570856000

1 -6.564525000 0.165306000 -3.427416000

1 -7.218488000 1.015321000 -1.998695000

1 -5.934997000 1.780743000 -2.983028000

6 -6.677151000 -1.837538000 -1.163708000

1 -7.155631000 -1.966290000 -0.169374000

1 -7.412567000 -1.351800000 -1.829122000

1 -6.485708000 -2.857570000 -1.558921000

6 -3.743784000 -0.227794000 3.319871000

6 -2.447630000 0.243514000 3.592603000

6 -4.590232000 0.491595000 2.459419000

1 -1.785971000 -0.329839000 4.261808000

1 -5.603998000 0.121633000 2.240931000

6 -1.967105000 1.431832000 3.005938000

6 -4.128284000 1.683178000 1.874396000

1 -4.776483000 2.251277000 1.188928000

6 -2.827711000 2.141761000 2.137174000

1 -2.470020000 3.067836000 1.660106000

1 -4.091901000 -1.167325000 3.777001000

6 -0.566813000 1.928908000 3.267798000

1 0.036226000 1.188307000 3.828919000

1 -0.043937000 2.140372000 2.312296000

1 -0.575548000 2.874538000 3.851160000

6 3.894245000 -0.226839000 4.962501000

6 4.560662000 -1.150422000 4.136518000

6 3.216868000 0.866498000 4.396242000

1 5.080672000 -2.011316000 4.588593000

1 2.691058000 1.589619000 5.039656000

6 4.564070000 -1.002845000 2.733554000

6 3.218517000 1.031077000 2.998227000

1 2.683631000 1.871867000 2.531962000

6 3.887697000 0.107704000 2.180474000

1 3.868618000 0.227387000 1.087503000

1 3.902186000 -0.365908000 6.055607000

6 5.212064000 -2.018404000 1.825574000

1 4.430897000 -2.504147000 1.200769000

1 5.930227000 -1.537817000 1.128262000

1 5.753460000 -2.798396000 2.397334000

108

## palladacycle 1 + Toluene on O – side

46 -0.068178000 1.060488000 -0.083105000

7 -0.081072000 -0.830007000 0.934847000

6 0.709248000 -1.219548000 1.931757000

6 0.628041000 -2.492468000 2.551295000

6 -0.319682000 -3.410866000 2.073857000

6 -1.168324000 -3.008443000 0.991572000

6 -1.021084000 -1.698490000 0.463003000

1 1.447599000 -0.471737000 2.263480000

6 -2.171806000 -3.856210000 0.404882000

6 -1.861751000 -1.243202000 -0.614481000

7 -1.670031000 0.028447000 -1.064547000

6 -2.832266000 -2.108421000 -1.193647000

6 -2.366146000 0.460308000 -2.116371000

6 -2.963340000 -3.430823000 -0.640663000

6 -3.587205000 -1.631526000 -2.313645000

1 -3.711211000 -4.117482000 -1.062044000

1 -2.309877000 -4.871427000 0.802373000

6 -3.319435000 -0.338020000 -2.799757000

1 -2.136709000 1.490298000 -2.439363000

6 1.412284000 1.970991000 0.852289000

8 2.215125000 1.436080000 1.657428000

7 1.546036000 3.304609000 0.519616000

8 0.668934000 3.796313000 -0.467755000

6 -0.183079000 2.799185000 -0.998901000

8 -0.887780000 3.175756000 -1.917427000

6 2.519454000 4.260202000 0.905597000

6 2.607330000 5.487391000 0.206442000

6 3.567759000 6.436940000 0.584043000

6 4.446708000 6.187929000 1.650979000

6 4.349766000 4.969282000 2.343997000

6 3.396519000 4.004736000 1.987706000

1 3.624782000 7.386970000 0.029586000

1 5.199104000 6.937254000 1.941315000

1 5.028101000 4.756973000 3.185419000

1 3.326065000 3.053291000 2.525255000

1 1.921464000 5.686504000 -0.625353000

8 3.071627000 -2.579369000 0.912589000

15 3.467626000 -1.556467000 -0.136745000

8 3.913499000 -0.068093000 0.329861000

1 3.232440000 0.441042000 0.881686000

6 4.945012000 -2.072794000 -1.068293000

6 5.385037000 -3.402020000 -0.911814000

6 5.629803000 -1.198691000 -1.937902000

6 6.502118000 -3.859136000 -1.630240000

6 6.745713000 -1.659930000 -2.653278000

6 7.180581000 -2.989830000 -2.502225000

1 4.842110000 -4.057805000 -0.213046000

1 5.293518000 -0.156133000 -2.045287000

1 6.848134000 -4.897616000 -1.506489000

1 7.283559000 -0.977673000 -3.330394000

1 8.056427000 -3.348502000 -3.065960000

6 2.148080000 -1.331909000 -1.385028000

6 1.297982000 -2.424358000 -1.652428000

6 1.998201000 -0.131315000 -2.112675000

6 0.325625000 -2.327777000 -2.660719000

6 1.026630000 -0.040766000 -3.125106000

6 0.199365000 -1.140963000 -3.405930000

1 1.402301000 -3.339520000 -1.049142000

1 2.650655000 0.726358000 -1.890338000

1 -0.340353000 -3.180919000 -2.863449000

1 0.902334000 0.901601000 -3.679986000

1 -0.562811000 -1.067205000 -4.196949000

6 1.604049000 -2.804551000 3.652841000

1 2.398676000 -3.476097000 3.266723000

1 2.106724000 -1.881807000 4.000356000

1 1.117464000 -3.285145000 4.525618000

6 -0.462752000 -4.790468000 2.656688000

1 0.262852000 -4.978024000 3.467304000

1 -1.483879000 -4.937251000 3.068429000

1 -0.306886000 -5.569783000 1.881110000

6 -3.969289000 0.216484000 -4.042853000

1 -3.736174000 -0.408389000 -4.931529000

1 -5.072550000 0.264080000 -3.946393000

1 -3.610967000 1.243325000 -4.244068000

6 -4.611829000 -2.526995000 -2.960080000

1 -5.346658000 -2.894106000 -2.213464000

1 -5.173514000 -2.011369000 -3.758904000

1 -4.134991000 -3.425561000 -3.407691000

6 -3.092672000 -2.581701000 4.070484000

6 -2.181118000 -1.533401000 4.286121000

6 -3.988497000 -2.530198000 2.988577000

1 -1.480995000 -1.584102000 5.136151000

1 -4.701637000 -3.351641000 2.817398000

6 -2.144730000 -0.412689000 3.429926000

6 -3.957897000 -1.425203000 2.121362000

1 -4.635637000 -1.375564000 1.254788000

6 -3.038316000 -0.388143000 2.337007000

1 -2.993762000 0.452991000 1.632024000

1 -3.104974000 -3.443503000 4.757014000

6 -1.200471000 0.739904000 3.671897000

1 -0.316985000 0.432068000 4.265375000

1 -0.839306000 1.173927000 2.716346000

1 -1.708025000 1.555818000 4.231438000

6 -5.144272000 2.788700000 -2.204455000

6 -4.144758000 3.171208000 -1.295936000

6 -6.111294000 1.835893000 -1.838081000

1 -3.375846000 3.899209000 -1.597208000

1 -6.900781000 1.541899000 -2.548146000

6 -4.103423000 2.629597000 0.008721000

6 -6.073422000 1.277084000 -0.549771000

1 -6.831930000 0.538655000 -0.243922000

6 -5.087000000 1.681125000 0.365907000

1 -5.091383000 1.264173000 1.384884000

1 -5.168731000 3.241370000 -3.208571000

6 -3.021112000 3.037861000 0.975265000

1 -2.691005000 4.081538000 0.805241000

1 -3.345814000 2.933879000 2.029279000

1 -2.114872000 2.399050000 0.838526000

123

## palladacycle 1 +Three toluene molecules

46 -0.758079000 0.372454000 -0.915377000

7 -0.060923000 -0.861796000 0.707363000

6 0.962826000 -0.637298000 1.529082000

6 1.308451000 -1.489726000 2.611987000

6 0.553493000 -2.657091000 2.811051000

6 -0.548170000 -2.912124000 1.931876000

6 -0.824701000 -1.977346000 0.898734000

1 1.537081000 0.282983000 1.340336000

6 -1.407091000 -4.061057000 2.045662000

6 -1.943769000 -2.179395000 0.015538000

7 -2.156323000 -1.244171000 -0.950942000

6 -2.790255000 -3.315675000 0.157649000

6 -3.187496000 -1.384681000 -1.780753000

6 -2.477794000 -4.256194000 1.199843000

6 -3.908035000 -3.451072000 -0.727859000

1 -3.110219000 -5.145860000 1.329502000

1 -1.207517000 -4.799674000 2.834960000

6 -4.106630000 -2.462175000 -1.710317000

1 -3.280119000 -0.596186000 -2.546436000

6 0.452057000 1.922197000 -0.771447000

8 1.546682000 1.972576000 -0.151530000

7 -0.026308000 3.050109000 -1.405790000

8 -1.154001000 2.877370000 -2.235265000

6 -1.586044000 1.518142000 -2.284545000

8 -2.440062000 1.296192000 -3.121021000

6 0.526764000 4.352963000 -1.500963000

6 0.045836000 5.243870000 -2.487045000

6 0.582765000 6.536833000 -2.577774000

6 1.593821000 6.957539000 -1.698338000

6 2.066909000 6.066733000 -0.719196000

6 1.542713000 4.771090000 -0.608369000

1 0.201061000 7.222362000 -3.350536000

1 2.011346000 7.973524000 -1.774157000

1 2.858311000 6.381731000 -0.021385000

1 1.932177000 4.075784000 0.141445000

1 -0.747872000 4.913495000 -3.168495000

8 3.512671000 -1.708097000 0.599057000

15 3.363358000 -1.224550000 -0.836986000

8 3.327803000 0.370737000 -1.127976000

1 2.581156000 0.893993000 -0.673971000

6 4.776389000 -1.721352000 -1.870129000

6 5.614651000 -2.745632000 -1.385860000

6 5.036435000 -1.126569000 -3.122192000

6 6.705084000 -3.180784000 -2.156420000

6 6.127979000 -1.564639000 -3.888008000

6 6.960353000 -2.592651000 -3.407743000

1 5.402065000 -3.179971000 -0.396341000

1 4.391250000 -0.312910000 -3.486686000

1 7.362496000 -3.979145000 -1.777313000

1 6.335370000 -1.097992000 -4.863905000

1 7.816271000 -2.933500000 -4.011589000

6 1.869592000 -1.923665000 -1.620934000

6 1.383133000 -3.160549000 -1.151582000

6 1.213043000 -1.266885000 -2.684393000

6 0.262925000 -3.749354000 -1.759034000

6 0.094005000 -1.862666000 -3.291745000

6 -0.375409000 -3.105227000 -2.834266000

1 1.881914000 -3.639786000 -0.294827000

1 1.582445000 -0.292408000 -3.036743000

1 -0.122145000 -4.710262000 -1.383507000

1 -0.426725000 -1.338891000 -4.107716000

1 -1.260597000 -3.563856000 -3.301959000

6 2.487672000 -1.111146000 3.463341000

1 3.369001000 -1.718488000 3.171409000

1 2.763900000 -0.054305000 3.301094000

1 2.293842000 -1.256896000 4.544292000

6 0.861614000 -3.627442000 3.920381000

1 1.773371000 -3.346033000 4.475163000

1 0.021824000 -3.681685000 4.646554000

1 1.013937000 -4.653622000 3.525042000

6 -5.252966000 -2.493918000 -2.691961000

1 -5.210741000 -3.388676000 -3.349194000

1 -6.235748000 -2.512943000 -2.176278000

1 -5.232782000 -1.598850000 -3.343192000

6 -4.834875000 -4.630570000 -0.587355000

1 -5.303910000 -4.651126000 0.419289000

1 -5.646108000 -4.619621000 -1.336781000

1 -4.283668000 -5.588109000 -0.700480000

6 -2.822220000 -1.274118000 3.722492000

6 -1.796079000 -0.319303000 3.836627000

6 -3.747990000 -1.194862000 2.668139000

1 -1.069381000 -0.389841000 4.662420000

1 -4.547843000 -1.945682000 2.573539000

6 -1.676129000 0.728214000 2.900433000

6 -3.635943000 -0.160544000 1.723802000

1 -4.339317000 -0.093518000 0.881326000

6 -2.606409000 0.784594000 1.840818000

1 -2.508218000 1.573583000 1.080522000

1 -2.894799000 -2.089935000 4.459386000

6 -0.586277000 1.765620000 3.004127000

1 0.235097000 1.443594000 3.673246000

1 -0.149007000 1.982052000 2.008210000

1 -0.981342000 2.726714000 3.400676000

6 -5.187682000 1.495201000 -1.380148000

6 -4.572120000 2.469253000 -0.573788000

6 -6.316181000 0.802916000 -0.912538000

1 -3.685490000 3.000193000 -0.955411000

1 -6.806262000 0.045550000 -1.544636000

6 -5.068235000 2.770358000 0.711049000

6 -6.824645000 1.094400000 0.367113000

1 -7.708849000 0.556788000 0.745448000

6 -6.205380000 2.066655000 1.167591000

1 -6.602343000 2.278306000 2.173979000

1 -4.766647000 1.301296000 -2.376785000

6 -4.386062000 3.785820000 1.595707000

1 -3.557699000 4.295565000 1.065159000

1 -5.097389000 4.561590000 1.949599000

1 -3.959086000 3.296588000 2.497815000

6 4.367231000 1.893271000 4.823215000

6 5.213317000 1.252031000 3.902243000

6 3.235116000 2.596987000 4.373303000

1 6.094381000 0.696856000 4.264727000

1 2.566049000 3.095182000 5.092414000

6 4.950114000 1.294141000 2.515285000

6 2.969641000 2.658019000 2.993419000

1 2.085083000 3.199783000 2.624267000

6 3.820178000 2.017252000 2.077601000

1 3.586989000 2.068634000 1.007097000

1 4.593464000 1.842305000 5.900567000

6 5.796589000 0.530329000 1.527388000

1 5.291915000 -0.433146000 1.291791000

1 5.897217000 1.073332000 0.566403000

1 6.807539000 0.314941000 1.927456000

# NCI analysis

The Non-Covalent Interaction analysis was carreid with the NCIPlots program from Contretas and coworkers (E. R. Johnson, S. Keinan, P. Mori-Sanchez, J. Contreras-Garcia, A. J. Cohen, and W. Yang, “Revealing Noncovalent Interactions”, **J. Am. Chem. Soc.** 132 , 6498 (2010); C. Narth, Z. Maroun, R. A. Boto, R. Chaudret, M-L Bonet, J-P Piquemal, J. Contreras-Garcia “A complete NCI perspective: from new bonds to reactivity” in the book “Applications of Topological Methods in Molecular Chemistry” Springer). The input wave-function file was generated using the uM06~GD3 /Def2TZVPP(H, C, P) + Def2TZVPPD(N,O) + SDD(Pd)+ ECP(SDD) model on the optimized geomtries (obtained with uBP86~GD3BJ /Def2SVP(H, C, O, N) + SDD(Pd)+ ECP(SDD). The obtained cube files were then ploted with the VMD program. Below the obtained isosurfaces for structures in Figure 3.


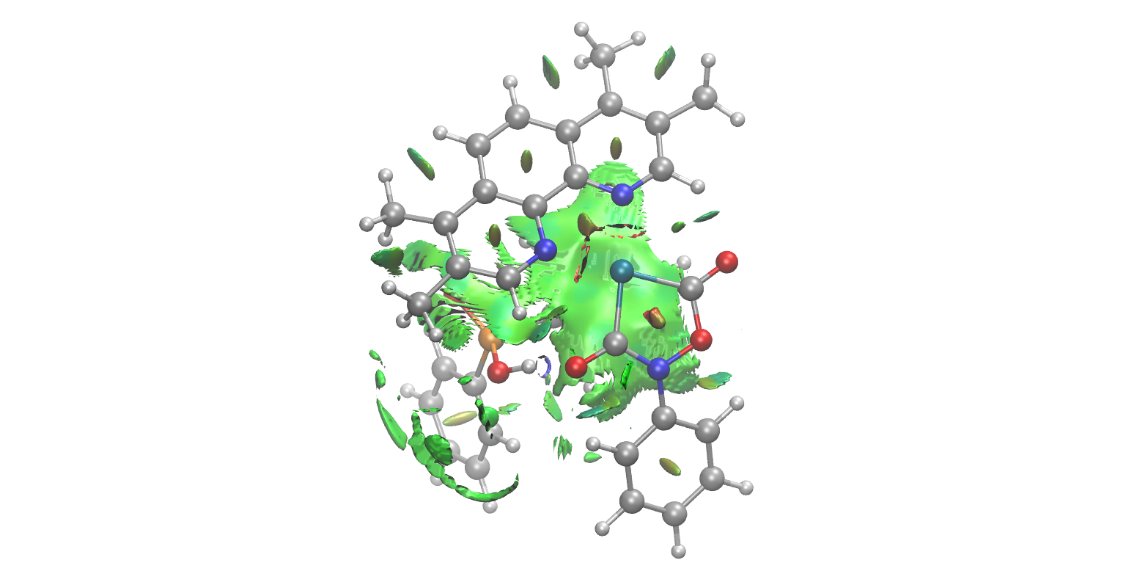


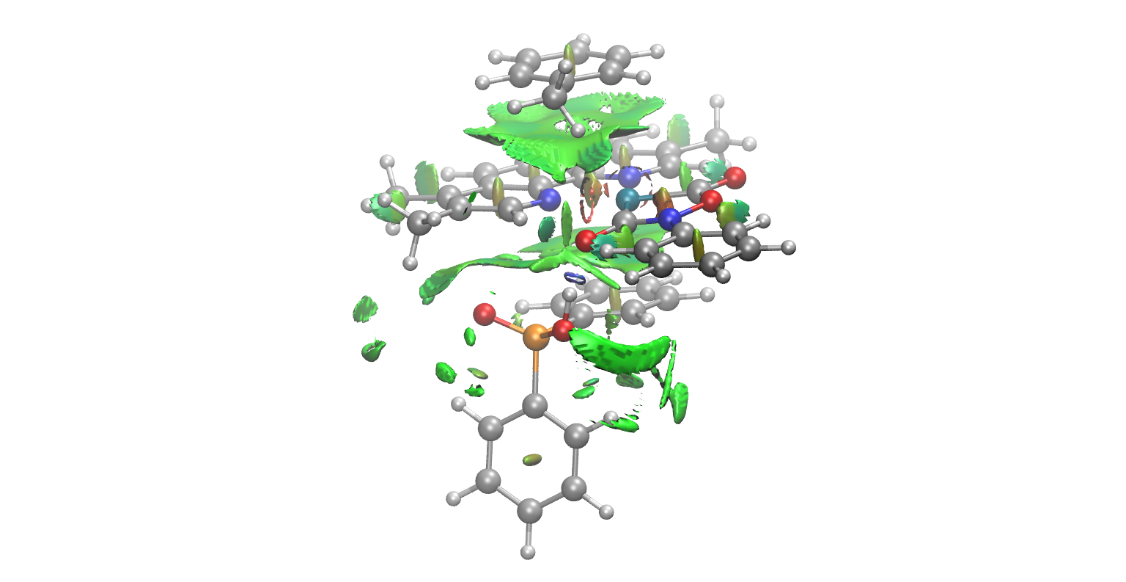

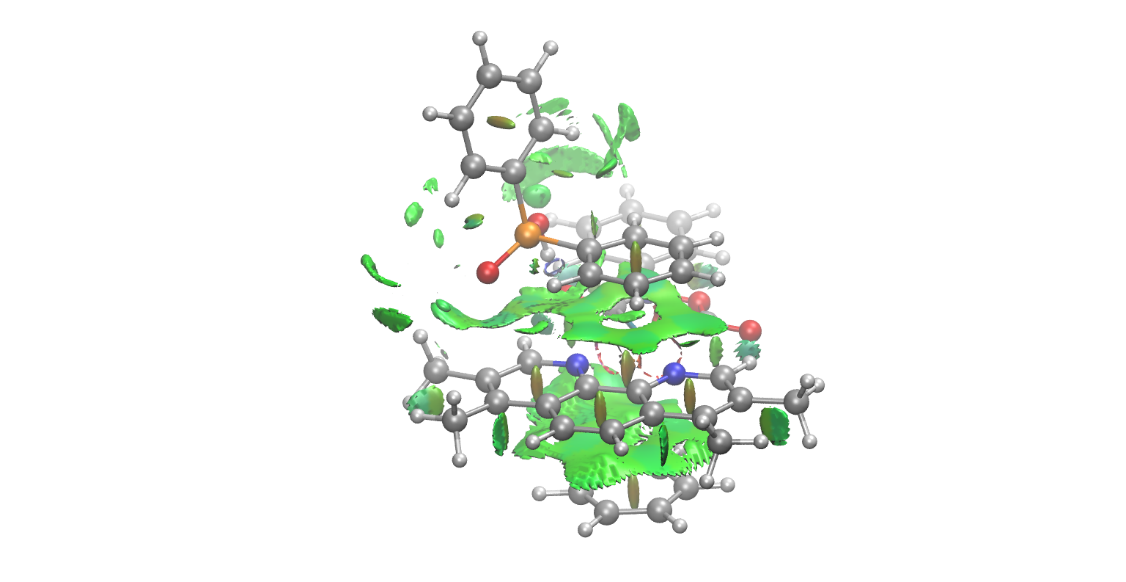


**Fig. S1** Additional NCI plots perspectives for the distorted structure (top) and for the tolene addition (both in the bottom).

# Energies

Table S2 Computed energies for conformational study, comparing different functionals and dispersion models. Absolute values in a.u. and relative values in kcal/mol.

|  | **Opt BP86 GD3BJ** | | **cam-b3lypNodisp** | | **cam-b3lyp** | | **M05noDisp** | |
| --- | --- | --- | --- | --- | --- | --- | --- | --- |
|  | Egas | Ggas | Esol | Gsol | Esol | Gsol | Esol | Gsol |
| **2** | -2242.5839 | -2242.1725 | -2243.3627 | -2242.9513 | -2243.4734 | -2243.0620 | -2242.9219 | -2242.9219 |
| **2_conf1** | -2242.5815 | -2242.1712 | -2243.3592 | -2242.9489 | -2243.4692 | -2243.0589 | -2242.9207 | -2242.9207 |
| **2_conf2** | -2242.5728 | -2242.1640 | -2243.3556 | -2242.9468 | -2243.4621 | -2243.0533 | -2242.9149 | -2242.9149 |
| **2_conf3** | -2242.5657 | -2242.1575 | -2243.3579 | -2242.9497 | -2243.4651 | -2243.0569 | -2242.9156 | -2242.9156 |
| **2_conf4** | -2242.5758 | -2242.1668 | -2243.373 | -2242.9640 | -2243.4679 | -2243.0589 | -2242.9321 | -2242.9321 |
|  | **M05** |  | **M06HFnoDisp** | | **M06HF** |  | **M11noDisp** | |
|  | Esol | Gsol | Esol | Gsol | Esol | Gsol | Esol | Gsol |
| **2** | -2242.9679 | -2242.5565 | -2243.7441 | -2243.3327 | -2243.758 | -2243.3466 | -2243.2483 | -2242.8369 |
| **2_conf1** | -2242.9664 | -2242.5561 | -2243.7378 | -2243.3275 | -2243.7515 | -2243.3412 | -2243.2448 | -2242.8345 |
| **2_conf2** | -2242.9575 | -2242.5487 | -2243.734 | -2243.3252 | -2243.7468 | -2243.3380 | -2243.2371 | -2242.8283 |
| **2_conf3** | -2242.9587 | -2242.5505 | -2243.7389 | -2243.3307 | -2243.7519 | -2243.3437 | -2243.24 | -2242.8318 |
| **2_conf4** | -2242.9691 | -2242.5601 | -2243.7377 | -2243.3287 | -2243.749 | -2243.3400 | -2243.2398 | -2242.8308 |
|  | **PW6B95D3** | | **tpssh** |  | **wbp97D** |  | **M06 no GD3** | |
|  | Esol | Gsol | Esol | Gsol | Esol | Gsol | Esol | Gsol |
| **2** | -2246.9596 | -2246.5482 | -2244.3523 | -2243.9409 | -2243.719 | -2243.3076 | -2243.0524 | -2242.6410 |
| **2_conf1** | -2246.9558 | -2246.5455 | -2244.3491 | -2243.9388 | -2243.7156 | -2243.3053 | -2243.0536 | -2242.6433 |
| **2_conf2** | -2246.947 | -2246.5382 | -2244.3463 | -2243.9375 | -2243.7073 | -2243.2985 | -2243.0571 | -2242.6483 |
| **2_conf3** | -2246.9497 | -2246.5415 | -2244.3488 | -2243.9406 | -2243.7097 | -2243.3015 | -2243.0617 | -2242.6535 |
| **2_conf4** | -2246.9508 | -2246.5418 | -2244.3632 | -2243.9542 | -2243.7118 | -2243.3028 | -2243.0632 | -2242.6542 |
|  | **M06 GD3** |  | **M15** |  | **B2PLYPD (only DFT)** | | **B2PLYPD (DFT+MP2)** | |
|  | Esol | Gsol | Esol | Gsol | Esol | Gsol | Esol | Gsol |
| **2** | -2243.0721 | -2242.6607 | -2240.2133 | -2239.8019 | -2242.6001 | -2242.1887 | -2241.8709 | -2241.4595 |
| **2_conf1** | -2243.0736 | -2242.6633 | -2240.2157 | -2239.8054 | -2242.6035 | -2242.1932 | -2241.8726 | -2241.4623 |
| **2_conf2** | -2243.074 | -2242.6652 | -2240.2245 | -2239.8157 | -2242.6009 | -2242.1921 | -2241.873 | -2241.4642 |
| **2_conf3** | -2243.0828 | -2242.6746 | -2240.2206 | -2239.8124 | -2242.6094 | -2242.2012 | -2241.8812 | -2241.4730 |
| **2_conf4** | -2243.0845 | -2242.6755 | -2240.2232 | -2239.8142 | -2242.6123 | -2242.2033 | -2241.8831 | -2241.4741 |

|  | **cam-b3lyp**  **Nodisp** | **cam-b3lyp** | **M05noDisp** | **M05** | **M06HF**  **noDisp** | **M06HF** | **M11noDisp** | **PW6B95D3** | **Tpssh noDisp** | **wbp97D** | **M06 noDisp** | **M06 Disp** | **M15noDisp** | **B2PLYPD** | **B2PLYPD (+ MP2)** |
| --- | --- | --- | --- | --- | --- | --- | --- | --- | --- | --- | --- | --- | --- | --- | --- |
| **2-conf_1_** | 3.07 | 3.46 | 1.71 | 1.90 | 4.68 | 4.84 | 3.08 | 3.26 | 2.88 | 3.00 | 0.29 | **0.12** | 0.41 | 1.00 | 0.00 |
| **2-conf_2_** | 5.42 | 7.90 | 5.32 | 7.32 | 7.13 | 7.84 | 7.81 | 8.64 | 4.72 | 8.12 | 1.53 | **0.12** | 5.35 | 0.76 | 0.00 |
| **2-conf_3_** | 8.84 | 10.93 | 9.74 | 11.50 | 9.10 | 9.65 | 10.91 | 11.88 | 8.13 | 11.50 | 0.59 | **0.24** | 1.76 | 0.59 | 0.00 |
| **2-conf_4_** | 1.11 | 10.43 | 1.17 | 6.49 | 10.91 | 12.44 | 12.14 | 12.33 | 0.75 | 11.39 | 0.82 | **0.12** | 1.35 | 0.00 | 0.00 |
| **Mean** | 4.61 | 8.18 | 4.48 | 6.80 | 7.96 | 8.69 | 8.48 | 9.03 | 4.12 | 8.50 | 0.81 | **0.15** | 2.22 | 0.59 | 0.00 |

Table S3 Heat map of the relative error (absolute values) for the relative electronic energies of the conformers of intermediate 2 (**conf1**, **conf2**, **conf3**, **conf4**) with respect intermediate **2**. The double-hybrid relative values between the conformers is used as reference value.

Table S4 Heat map of the relative error (absolute values) for the relative free energies of the conformers of intermediate 2 (**conf1**, **conf2**, **conf3**, **conf4**) with respect intermediate **2**. The double-hybrid relative values between the conformers is used as reference value.

|  | **cam-b3lyp**  **Nodisp** | **cam-b3lyp** | **M05noDisp** | **M05** | **M06HF**  **noDisp** | **M06HF** | **M11noDisp** | **PW6B95D3** | **Tpssh noDisp** | **wbp97D** | **M06**  **noDisp** | **M06 Disp** | **M15noDisp** | **B2PLYPD** | **B2PLYPD (+ MP2)** |
| --- | --- | --- | --- | --- | --- | --- | --- | --- | --- | --- | --- | --- | --- | --- | --- |
| **2-conf_1_** | 1.86 | 2.10 | 1.43 | 1.15 | 2.84 | 2.94 | 1.87 | 1.98 | 1.75 | 1.82 | 0.18 | **0.07** | 0.25 | 0.61 | 0.00 |
| **2-conf_2_** | 3.29 | 4.79 | 4.16 | 4.45 | 4.33 | 4.76 | 4.74 | 5.24 | 2.87 | 4.93 | 0.93 | **0.07** | 3.25 | 0.46 | 0.00 |
| **2-conf_3_** | 5.37 | 6.64 | 7.05 | 6.98 | 5.53 | 5.86 | 6.62 | 7.22 | 4.93 | 6.98 | 0.36 | **0.14** | 1.07 | 0.36 | 0.00 |
| **2-conf_4_** | 0.68 | 6.33 | 1.57 | 3.94 | 6.63 | 7.55 | 7.37 | 7.49 | 0.46 | 6.91 | 0.50 | **0.07** | 0.82 | 0.00 | 0.00 |
| **Avg Error** | 2.80 | 4.97 | 3.55 | 4.13 | 4.83 | 5.28 | 5.15 | 5.48 | 2.50 | 5.16 | 0.49 | **0.09** | 1.35 | 0.36 | 0.00 |

# RMSD analysis

The RMSD was calculated by means of the ChemCraft Structure Compared tool. It is described in <https://www.chemcraftprog.com/help/structcomparerwindow.html>,^1^ which includes the algorithm:

Before printing the RMSDs, the program rotates and translates one of the structures to minimize the RMSD value. In current implementation the rotation is made iteratively, with minimal step of 1×10⁻¹⁸ radians. The error in RMSD caused by discrete rotation is approximately 1×10⁻¹⁴ Å for molecules of 50–100 atoms.

To quantify structural similarity, the Root-Mean-Square Deviation (RMSD) of the atomic coordinates was computed using the **Structures Comparer** utility in Chemcraft. The algorithm applies a coordinate transformation that translates and rotates one molecule relative to the other to minimize the spatial distance between corresponding atom pairs. The final minimized RMSD for all $N$ atoms is calculated using the standard formula:

$$RMSD= \sqrt{\frac{1}{N}\sum_{i=1}^{N} \left| x_{i}-x_{i}^{ref} \right|^{2}}$$

$$RMSD=\surd\{\backslash frac\{1\}\{N\}\sum\_\{i=1\}^\{N\}|x\_\{i\}-x\_\{i\}^\{ref\}|^\{2\}\})$$

where $x_{i}$ and $x_{i}^{ref}$ represent the optimized, superimposed Cartesian coordinates of atom $i$ in the target and reference structures, respectively.

Relevant RMSD computed values

| ORCA BJ vs GD4 |  | |  | |  | |
| --- | --- | --- | --- | --- | --- | --- |
| **Atom** | **RMSD, Å** | **Atom** | | **RMSD, Å** | |  |
| Pd1 | 0.070 | C31 | | 0.143 | |  |
| N2 | 0.005 | C32 | | 0.171 | |  |
| C3 | 0.047 | C33 | | 0.126 | |  |
| C4 | 0.043 | C34 | | 0.065 | |  |
| C5 | 0.052 | C35 | | 0.058 | |  |
| C6 | 0.064 | H36 | | 0.231 | |  |
| C7 | 0.067 | H37 | | 0.148 | |  |
| H8 | 0.042 | H38 | | 0.056 | |  |
| H9 | 0.036 | H39 | | 0.088 | |  |
| H10 | 0.052 | H40 | | 0.178 | |  |
| C11 | 0.073 | C41 | | 0.107 | |  |
| C12 | 0.076 | C42 | | 0.255 | |  |
| N13 | 0.077 | C43 | | 0.149 | |  |
| C14 | 0.084 | C44 | | 0.360 | |  |
| C15 | 0.086 | C45 | | 0.295 | |  |
| C16 | 0.083 | C46 | | 0.373 | |  |
| C17 | 0.093 | H47 | | 0.331 | |  |
| H18 | 0.090 | H48 | | 0.178 | |  |
| H19 | 0.073 | H49 | | 0.473 | |  |
| C20 | 0.094 | H50 | | 0.381 | |  |
| H21 | 0.089 | H51 | | 0.494 | |  |
| H22 | 0.101 | H52 | | 0.112 | |  |
| H23 | 0.099 | All H atoms | | 0.221 | |  |
| C24 | 0.062 | All C atoms | | 0.153 | |  |
| O25 | 0.075 | All N atoms | | 0.069 | |  |
| N26 | 0.068 | All O atoms | | 0.090 | |  |
| O27 | 0.090 | All Pd atoms | | 0.070 | |  |
| C28 | 0.063 | All atoms | | 0.175 | |  |
| O29 | 0.103 | With weights | | 0.175 | |  |
| C30 | 0.078 | **RMSD** | | **0.175** | |  |

| BJ ORCA vs Gaussian 16 |  | |  | |  | |
| --- | --- | --- | --- | --- | --- | --- |
| **Atom** | **RMSD, Å** |  | |  | |  |
| Pd1 | 0.014 | C31 | | 0.013 | |  |
| N2 | 0.009 | C32 | | 0.020 | |  |
| C3 | 0.008 | C33 | | 0.018 | |  |
| C4 | 0.014 | C34 | | 0.008 | |  |
| C5 | 0.016 | C35 | | 0.003 | |  |
| C6 | 0.012 | H36 | | 0.027 | |  |
| C7 | 0.009 | H37 | | 0.024 | |  |
| H8 | 0.008 | H38 | | 0.006 | |  |
| H9 | 0.018 | H39 | | 0.009 | |  |
| H10 | 0.024 | H40 | | 0.015 | |  |
| C11 | 0.015 | C41 | | 0.013 | |  |
| C12 | 0.010 | C42 | | 0.032 | |  |
| N13 | 0.012 | C43 | | 0.019 | |  |
| C14 | 0.010 | C44 | | 0.039 | |  |
| C15 | 0.011 | C45 | | 0.033 | |  |
| C16 | 0.013 | C46 | | 0.038 | |  |
| C17 | 0.011 | H47 | | 0.046 | |  |
| H18 | 0.017 | H48 | | 0.025 | |  |
| H19 | 0.020 | H49 | | 0.054 | |  |
| C20 | 0.010 | H50 | | 0.043 | |  |
| H21 | 0.010 | H51 | | 0.048 | |  |
| H22 | 0.012 | H52 | | 0.013 | |  |
| H23 | 0.015 | All H atoms | | 0.027 | |  |
| C24 | 0.006 | All C atoms | | 0.018 | |  |
| O25 | 0.009 | All N atoms | | 0.010 | |  |
| N26 | 0.007 | All O atoms | | 0.018 | |  |
| O27 | 0.015 | All Pd atoms | | 0.014 | |  |
| C28 | 0.014 | All atoms | | 0.021 | |  |
| O29 | 0.025 | With weights | | 0.021 | |  |
| C30 | 0.005 | **RMSD** | | **0.021** | |  |

**References**

[1] Zhurko GA, Zhurko DA. Chemcraft – graphical software for visualization of quantum chemistry computations. Available at: [https://chemcraftprog.com/](https://chemcraftprog.com/?utm_source=chatgpt.com)
